# Global, Regional, and National Burdens of Refraction Disorders in Children and Adolescents From 2010 to 2021

**DOI:** 10.1155/joph/5159332

**Published:** 2026-05-20

**Authors:** Jiayu Zhao, Lili Sun, Xiao Guo, Lijun Zhang

**Affiliations:** ^1^ Department of Ophthalmology, Dalian Medical University, Dalian, 116000, Liaoning, China, dlmedu.edu.cn; ^2^ Department of Ophthalmology, the Third Affiliated Hospital of Jinzhou Medical University, Jinzhou, 121000, Liaoning, China, jzmu.edu.cn; ^3^ Zhongshan Ophthalmic Center, State Key Laboratory of Ophthalmology, Sun Yatsen University, Guangzhou, 510000, Guangdong, China; ^4^ Department of Ophthalmology, Affiliated Dalian Third People’s Hospital of Dalian Medical University, Dalian, 116000, Liaoning, China

**Keywords:** Global Burden of Disease, refraction disorders, vision loss

## Abstract

**Objective:**

This analysis investigates the trends in prevalence and disability attributable to refractive disorders among children and adolescents worldwide from 2010 to 2021, using data from the Global Burden of Disease (GBD) 2021 study.

**Materials and Methods:**

Data on prevalence and years lived with disability (YLDs) were extracted from the GBD 2021 repository. The sociodemographic index (SDI) was derived from World Bank datasets. Analytical methods included the Wilcoxon signed‐rank test, Kruskal–Wallis test, and linear regression.

**Results:**

Between 2010 and 2021, a modest global decline was observed in both the prevalence and YLD rates of refractive disorders, with estimated annual percentage changes (EAPCs) of −0.193 and −0.191, respectively. The burden was consistently greater in females compared to males. The most pronounced decrease occurred in the 15–19 age cohorts. Contrarily, an upward trend was noted in the majority of nations and regions. Tropical Latin America recorded the highest YLD rates, whereas East Asia experienced the most rapid increase. Regions with middle‐high SDI demonstrated a significant rise in both prevalence and YLDs.

**Conclusion:**

Although the global burden of refractive disorders diminished over the study period, especially among older adolescents, disparities persisted. Females carried a disproportionately higher burden, and middle‐high SDI regions exhibited an increasing trend, underscoring the necessity for targeted public health strategies in vision care.

## 1. Background

Refractive disorders represent a predominant cause of visual impairment and blindness globally. In 2020, 157 million people suffered from moderate to severe visual impairment due to refractive disorders, including approximately 12.8 million children [1, 2]. The extended lifespan of children places them at risk for lifelong visual disability if refractive conditions are not adequately managed. Uncorrected vision can lead to misdiagnosis of learning disabilities, adversely affect academic performance, and limit future socioeconomic opportunities, thereby imposing costs on both individuals and societies [3]. The global economic impact is substantial, with productivity losses from uncorrected myopia alone estimated at $244 billion, disproportionately affecting low‐ and middle‐income countries with limited access to eye care services [4].

International efforts to combat this issue have been robust. The World Health Organization (WHO) set a target in 2021 to increase effective refractive error coverage (eREC) by 40% by 2030 and subsequently launched the SPECS 2030 initiative to promote accessible, high‐quality refractive care. Concurrently, the Global Burden of Disease (GBD) database has been refined, incorporating more comprehensive data to improve the accuracy of burden estimates for conditions like refractive disorders [5].

In the past decade, many countries and regions have experienced changes in lifestyle and visual habits. The COVID‐19 pandemic, in particular, accelerated the digital transformation of consumption scenarios. The rapid growth of remote work, online education, virtual meetings, and e‐commerce has led to reduced outdoor activity, sedentary behavior, and unhealthy eating habits [6]. Although data from the GBD 2019 report indicate that the burden of visual impairment due to refractive disorders among children and adolescents under 20 has shown a downward trend for nearly 30 years, it remains unclear whether this trend has changed since 2019 [5].

Moreover, recent analyses utilizing the GBD 2021 dataset have provided crucial updates on the global epidemiology of refractive disorders, confirming a general decline in age‐standardized prevalence and years lived with disability (YLDs) rates from 1990 to 2021, while highlighting persistent disparities related to sex, age, and sociodemographic index (SDI) [7–9]. Notably, studies have begun to dissect the burden in specific age groups, revealing that population growth is a primary driver of increasing case numbers, with distinct patterns between children/adolescents and the elderly, and indicating a rising burden in high SDI regions among the younger population [10]. However, a comprehensive, comparative analysis focused exclusively on the pediatric and adolescent population (0–19 years) over the recent 2010–2021 period—encompassing the critical pre‐ and early‐pandemic years—remains lacking. Therefore, we collaborated with the GBD to analyze the trends in the burden of refractive disorders among children and adolescents from 2010 to 2021 at global, regional, and national levels. The novel contributions of this analysis lie in: (1) providing a detailed, updated assessment of sex and age disparities in the burden of refractive disorders, with a specific focus on trends in the 15–19 age groups; (2) investigating the emerging and contrasting trends across SDI regions, particularly identifying the increasing burden in middle‐high SDI regions—a finding not previously highlighted; and (3) offering a comprehensive national‐level mapping of prevalence, YLD rates, and their estimated annual percentage changes (EAPCs) for 204 countries, thereby identifying the countries with the most significant increases and decreases. This granular, multidimensional analysis may provide more targeted and equitable public health strategies for vision care in the postpandemic era.

## 2. Methods

### 2.1. Data Extraction

Data were obtained from the Global Health Data Exchange (GHDx) for the GBD 2021 study, which provides estimates for 369 diseases and injuries across 204 countries and 21 regions. We extracted data on refractive disorders, specifically focusing on refractive disorders from 2010 to 2021 [11]. The included data comprised the following: (1) global, age‐specific, and sex‐specific prevalence and YLD counts and rates (per 100,000) from 2010 to 2021; (2) crude YLD rates for GBD regions in 2010 and 2021; and (3) the SDI for all included countries in 2021. Complementary data on mobile cellular subscriptions, urbanization, GDP per capita, electricity access, and fertility rates were sourced from the World Bank for 2021. Ethical approval was waived as the data are publicly available.

### 2.2. Variables

YLDs assess the cumulative impact of specific health conditions by estimating the time individuals live with a less‐than‐ideal health status, excluding the risk of mortality [12]. Previous studies have indicated that the burden of refractive disorders, a nonfatal condition, can be effectively represented using YLDs [13]. The 2021 GBD’s SDI reflects overall development, encompassing total fertility rates, income distribution disparities, and educational levels. The SDI ranges from 0 to 1, with classifications as follows: high SDI (> 0.81), middle‐high SDI (0.70–0.81), middle SDI (0.61–0.69), middle‐low SDI (0.46–0.60), and low SDI (< 0.46).

According to the ICD‐10 classification, refractive disorders include the following: (1) myopia: difficulty seeing distant objects clearly while nearby objects are clear; (2) hyperopia: difficulty seeing nearby objects clearly while distant objects are relatively clear; (3) astigmatism: blurred or distorted vision due to irregular shapes of the cornea or lens, affecting both near and distant objects; (4) accommodation disorders: difficulty adjusting focus, commonly experienced during near reading; (5) visual fatigue: discomfort in the eyes after prolonged use, often accompanied by blurred vision and headaches; (6) other refractive errors: additional refractive issues resulting from specific pathological or physiological causes; and (7) visual impairment: partial or complete loss of vision due to various reasons [14].

### 2.3. Statistical Analyses

Outcomes are reported as rates per 100,000 population with 95% uncertainty intervals (UIs). The Wilcoxon signed‐rank test [15] was used for paired comparisons (e.g., by sex), and the Kruskal–Wallis test [16] assessed differences across SDI regions, with post hoc Bonferroni correction. Linear regression was used to evaluate the association between SDI and outcome rates, and the standard diagnostics for the linear regression models were conducted. The assumptions of linearity and homoscedasticity of residuals were visually assessed and were deemed acceptable. Additionally, to aid in the interpretation of the models, the coefficient of determination (*R* ^2^) for each regression was calculated and reported in the Supporting Information. All data analyses were conducted within the RStudio (v4.0.4) environment, applying a significance level of *p* < 0.05.

## 3. Results

### 3.1. Global Trends and Global Trends by Sex and Age

Globally, the prevalence and YLD rates for refractive disorders exhibited a slight decline from 2010 to 2021, with EAPCs of −0.193 (95% UI: −0.269 to −0.117) and −0.191 (95% UI: −0.273 to −0.110), respectively (Table 1). Females consistently bore a higher burden than males. Moreover, the decline in prevalence and YLD rates from 2010 to 2021 was greater in females than in males, with the EAPC for prevalence rates at −0.221 (female) (95% UI: −0.237‐0.091) and −0.164 (male) (95% UI: −0.252‐0.069). The YLD rates had EAPCs of −0.221 (95% UI: −0.293 to −0.148) for females and −0.160 (95% UI: −0.252 to −0.069) for males. By age group, both prevalence and YLD rates exhibited a downward trend, with the 15–19 age group showing the most significant declines, having EAPCs of −0.338 (95% UI: −0.399 to −0.277) for prevalence rates and −0.335 (95% UI: −0.396 to −0.274) for YLD rates. Stratification by SDI revealed declining trends in high and low‐middle SDI regions but an increasing trend in middle‐high SDI regions.

**TABLE 1:** Prevalence rate of refractive disorders and average annual percentage changes from 2010 to 2021 at the global and regional levels.

	Prevalence rate				
	Case (*n*), 2010	Prevalence, 2010 (per 100,000 population)	Case (*n*), 2021	Prevalence, 2021 (per 100,000 population)	EAPC 2010–2021
Worldwide	23,626,575.0 (19,066,839.7–28,499,915.6)	951.3 (767.7–1147.6)	24,453,843.4 (19,728,778.7–29,660,612.4)	927.7 (748.5–1125.3)	−0.193 (−0.269‐0.117)
Sex					
Male	11,385,475.0 (9,150,344.5–13,759,455.6)	888.6 (714.2–1073.9)	11,811,834.9 (9,498,962.8–14,333,313.3)	869.5 (699.2–1055.1)	−0.164 (−0.237‐0.091)
Female	12,241,099.9 (9,911,955.9–14,772,725.0)	1018.1 (824.4–1228.7)	12,642,008.5 (10,193,460.1–15,312,382.9)	989.7 (798.0–1198.8)	−0.221 (−0.300‐0.141)
Age group					
< 5	3,251,798.9 (2,586,514.8–3,989,277.2)	496.1 (394.6–608.6)	3,257,391.9 (2,585,916.3–3,998,273.7)	494.9 (392.9–607.5)	−0.044 (−0.074‐0.014)
5–9	6,149,337.4 (4,583,272.9–7,936,889.3)	1001.3 (746.3–1292.3)	6,715,226.7 (4,998,175.8–8,672,845.9)	977.4 (727.5–1262.3)	−0.157 (−0.211‐0.103)
10–14	7,021,388.2 (5,235,390.8–8,910,484.6)	1162.7 (866.9–1475.5)	7,425,840.0 (5,531,335.5–9,462,282.4)	1113.9 (829.7–1419.4)	−0.329 (−0.414‐0.243)
15–19	7,204,050.4 (5,553,519.2–8,882,579.6)	1181.1 (910.5–1456.3)	7,055,384.8 (5,431,243.4–8,764,702.7)	1130.7 (870.4–1404.6)	−0.338 (−0.399‐0.277)
Sociodemographic index					
High	2,509,252.2 (2,030,404.4–3,011,599.3)	1048.9 (848.7–1258.9)	2,391,728.3 (1,945,361.7–2,894,592.5)	1027.7 (835.9–1243.8)	−0.212 (−0.271‐0.153)
Middle‐high	3,060,321.9 (2,497,851.6–3,682,345.4)	1024.7 (836.4–1233.0)	3,117,692.9 (2,525,245.2–3,781,650.2)	1027.7 (832.4–1246.6)	0.122 (0.008–0.237)
Middle	7,786,390.2 (6,304,576.6–9,391,151.2)	1048.7 (849.1–1264.8)	7,765,298.2 (6,258,989.0–9,415,937.7)	1036.5 (835.4–1256.8)	−0.047 (−0.131–0.037)
Low‐middle	7,176,578.7 (5,767,883.8–8,687,548.5)	980.1 (787.7–1186.5)	7,337,908.7 (5,896,608.7–8,956,770.7)	960.0 (771.4–1171.8)	−0.195 (−0.271‐0.153)
Low	3,075,360.9 (2,463,352.8–3,734,319.9)	655.7 (525.2–796.2)	3,822,223.5 (3,053,290.8–4,637,018.8)	654.3 (522.6–793.7)	0.002 (−0.043–0.046)
Region					
Andean Latin America	293,746.3 (238,643.9–3,53,282.6)	1338.9 (1087.7–1610.2)	313,555.1 (254,631.3–3,79,397.6)	1324.5 (1075.6–1602.6)	−0.137 (−0.175‐0.100)
Australasia	92,832.5 (74,742.8–1,13,216.2)	1348.8 (1085.9–1644.9)	98,509.8 (79,915.0–121,915.5)	1306.2 (1059.6–1616.5)	−0.183 (−0.355‐0.010)
Caribbean	155,327.8 (125,109.1–189,587.6)	996.6 (802.7–1216.4)	147,134.5 (117,586.1–179,099.2)	964.0 (770.4–1173.5)	−0.367 (−0.437‐0.298)
Central Asia	273,326.0 (222,129.1–329,996.0)	892.3 (725.2–1077.3)	300,446.5 (242,817.6–36,6522.1)	867.7 (701.3–1058.6)	−0.233 (−0.334‐0.132)
Central Europe	169,734.8 (138,660.2–206,155.4)	655.4 (535.4–796.0)	155,708.3 (126,486.9–189,663.9)	661.0 (536.9–805.1)	0.078 (0.034–0.123)
Central Latin America	964,551.3 (790,680.0–1,150,727.3)	1050.9 (861.5–1253.8)	943,231.8 (767,218.2–1,129,780.8)	1106.0 (899.6–1324.7)	0.549 (0.340–0.759)
Central Sub‐Saharan Africa	244,115.4 (190,669.7–301,517.1)	437.5 (341.7–540.4)	343,444.3 (267,234.9–425,739.6)	466.9 (363.3–578.8)	0.496 (0.386–0.606)
East Asia	2,497,273.5 (2,022,398.6–3,028,378.1)	745.5 (603.7–904.1)	2,624,930.8 (2,120,635.3–3,201,298.0)	761.0 (614.8–928.0)	0.624 (0.202–1.048)
Eastern Europe	469,279.7 (384,017.8–563,921.4)	1048.9 (858.3–1260.4)	500,339.1 (405,865.6–608,431.0)	1083.9 (879.3–1318.1)	0.335 (0.192–0.478)
Eastern Sub‐Saharan Africa	916,077.7 (731,975.1–1,112,746.2)	492.6 (393.6–598.4)	1,113,054.0 (890,983.7–1,350,857.1)	489.1 (391.5–593.6)	−0.046 (−0.105–0.013)
High‐income Asia Pacific	365,334.5 (295,991.8–440,524.0)	1004.8 (814.1–1211.6)	308,338.0 (249,450.9–372,606.6)	1001.5 (810.2–1210.2)	−0.022 (−0.126–0.082)
High‐income North America	930,289.4 (752,910.6–1,126,024.4)	1012.9 (819.8–1226.1)	851,754.3 (686,628.6–1,034,642.5)	951.1 (766.7–1155.3)	−0.679 (−0.788‐0.569)
The Middle East and North Africa	2,986,002.7 (2,429,472.6–3,608,014.2)	1396.1 (1135.9–1686.9)	3,304,554.7 (2,674,019.7–4,026,860.9)	1397.3 (1130.7–1702.7)	−0.001 (−0.105–0.103)
Oceania	49,116.2 (39,714.3–59,457.5)	962.3 (778.1–1164.9)	61,967.2 (49,895.2–75,067.3)	970.3 (781.3–1175.4)	0.057 (0.024–0.090)
South Asia	6,389,017.9 (5,077,042.2–7,820,641.0)	935.9 (743.7–1145.6)	6,316,165.6 (4,979,367.8–7,721,653.7)	924.1 (728.5–1129.7)	−0.081 (−0.245–0.084)
Southeast Asia	2,802,358.3 (2,264,043.1–3,372,824.9)	1208.5 (976.3–1454.5)	2,764,212.3 (2,221,824.0–3,351,524.4)	1205.7 (969.1–1461.8)	−0.026 (−0.062–0.009)
Southern Latin America	306,195.1 (248,554.3–370,978.7)	1514.9 (1229.7–1835.4)	297,515.8 (239,452.9–362,533.0)	1525.0 (1227.4–1858.2)	0.015 (−0.130–0.160)
Southern Sub‐Saharan Africa	195,501.0 (156,185.3–238,683.3)	649.6 (519.0–793.1)	205,370.9 (163,585.1–250,946.6)	656.9 (523.2–802.7)	0.111 (0.082–0.141)
Tropical Latin America	1,295,411.5 (1,057,597.6–1,563,001.6)	1861.8 (1520.0–2246.4)	1,106,200.8 (899,166.1–1,334,219.4)	1661.3 (1350.4–2003.7)	−1.329 (−1.892‐0.763)
Western Europe	1,142,026.6 (923,503.1–1,375,517.6)	1245.7 (1007.3–1500.4)	1,146,511.0 (923,062.6–1,383,499.5)	1250.1 (1006.5–1508.5)	0.016 (−0.021–0.053)
Western Sub‐Saharan Africa	1,089,056.8 (883,202.7–1,300,784.5)	555.5 (450.5–663.5)	1,550,898.6 (1,249,500.5–1,873,845.9)	577.5 (465.2–697.7)	0.422 (0.288–0.556)

### 3.2. Burden Trends by Region

Eight of the 21 GBD regions showed an increasing prevalence trend (Figure 1, Table 1), and seven regions saw an increase in the YLDs rate (Table 2). Tropical Latin America had the highest YLD rates in both 2010 (67.1 per 100,000 population) and 2021 (59.4 per 100,000 population), whereas Central Sub‐Saharan Africa had the lowest YLD rates in 2010 (14.6 per 100,000 population) and 2021 (15.7 per 100,000 population). Additionally, East Asia showed the largest increase in prevalence and YLD rates between 2010 and 2021, with EAPCs of 0.624 (95% UI: 0.202–1.048) and 0.808 (95% UI: 0.383–1.236), respectively (Tables 1 and 2). Conversely, Tropical Latin America had the most significant decline, with EAPCs of −1.329 (95% UI: −1.892 to −0.763) for prevalence and −0.1403 (95% UI: −1.928 to −0.875) for YLDs.

FIGURE 1Burden of refraction disorders in global and 21 regions in 2010 and 2021. (a) Prevalent rates of burden of refraction disorders in 2010. (b) Prevalent rate of burden of refraction disorders in 2021. (c) YLD rates of burden of refraction disorders in 2010. (d) YLD rates of burden of refraction disorders in 2021. YLDs = years lived with disability.

**Figure:**
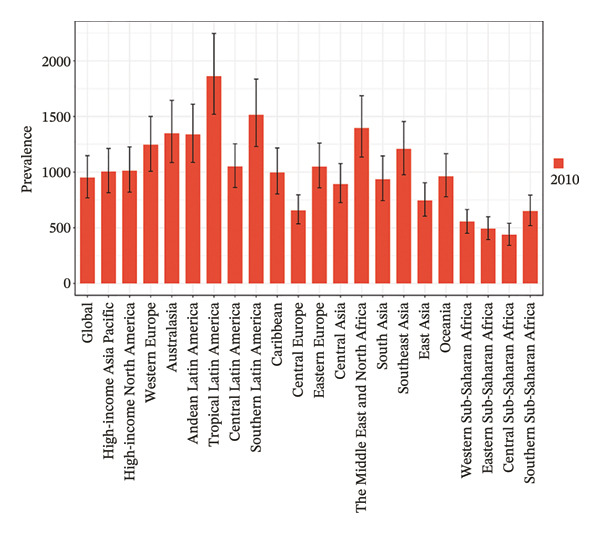
(a)

**Figure:**
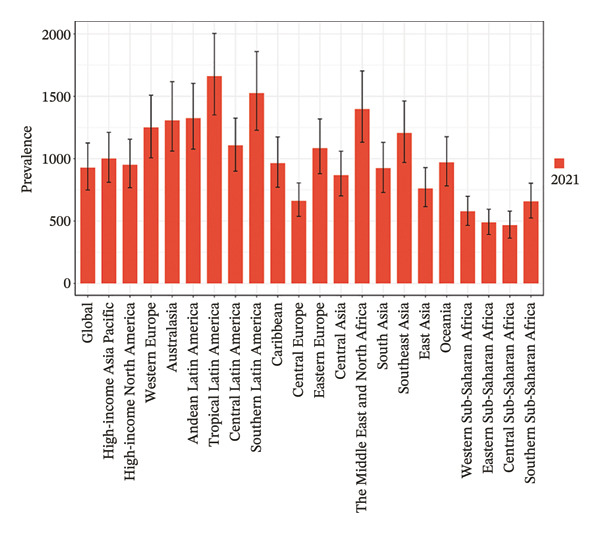
(b)

**Figure:**
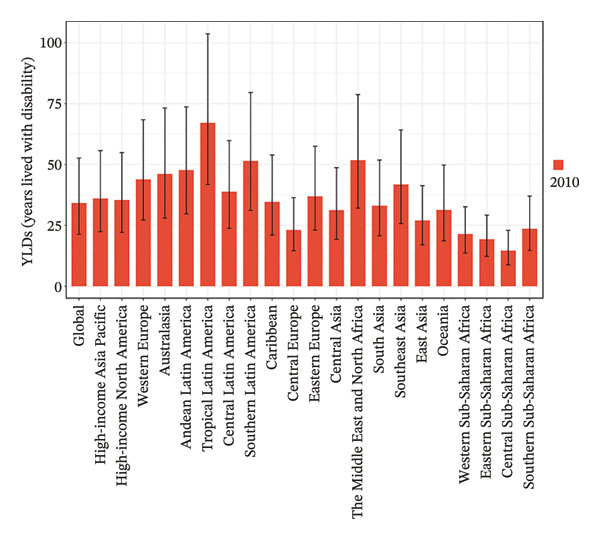
(c)

**Figure:**
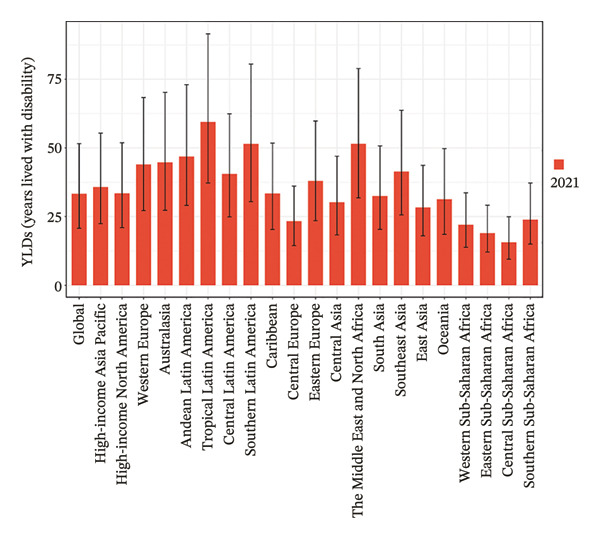
(d)

**TABLE 2:** Years lived with disability of refractive disorders and average annual percentage changes from 2010 to 2021 at the global and regional levels.

	YLDs rate				
	Case (*n*), 2010	YLDs, 2010 (per 100,000 population)	Case (*n*), 2021	YLDs, 2021 (per 100,000 population)	EAPC 2010–2021
Worldwide	848,397.4 (528,504.0–130,7387.3)	34.2 (21.3–52.6)	878,104.2 (546,921.2–135,8697.8)	33.3 (20.7–51.5)	−0.191 (−0.273‐0.110)
Sex					
Male	407,507.6 (253,516.7–627,716.0)	31.8 (19.8–49.0)	422,663.3 (262,486.6–654,361.7)	31.1 (19.3–48.2)	−0.160 (−0.252‐0.069)
Female	440,889.8 (274,987.3–679,671.2)	36.7 (22.9–56.5)	455,440.9 (284,510.8–704,435.5)	35.7 (22.3–55.1)	−0.221 (−0.293‐0.148)
Age group					
< 5	109,203.1 (66,985.6–178,523.0)	16.7 (10.2–27.2)	109,613.9 (67,600.2–180,490.9)	16.7 (10.3–27.4)	−0.017 (−0.043–0.008)
5–9	215,912.1 (130,852.8–352,798.3)	35.2 (21.3–57.4)	235,793.6 (142,175.3–386,695.8)	34.3 (20.7–56.3)	−0.154 (−0.212‐0.097)
10–14	254,223.8 (155,884.2–388,311.6)	42.1 (25.8–64.3)	269,043.2 (165,231.9–411,917.6)	40.4 (24.8–61.8)	−0.320 (−0.404‐0.235)
15–19	269,058.4 (167,446.0–411,687.2)	44.1 (27.5–67.5)	263,653.5 (163,373.4–403,187.6)	42.3 (26.2–64.6)	−0.335 (−0.396‐0.274)
Sociodemographic index					
High	88,554.3 (54,871.4–136,610.7)	37.0 (22.9–57.1)	84,474.0 (52,644.6–130,361.5)	36.3 (22.6–56.0)	−0.190 (−0.242‐0.137)
Middle‐high	108,276.2 (66,847.2–167,824.8)	36.3 (22.4–56.2)	111,042.8 (68,858.9–172,609.4)	36.6 (22.7–56.9)	0.173 (0.047–0.299)
Middle	280,677.9 (174,956.3–431,559.8)	37.8 (23.6–58.1)	279,794.4 (174,911.6–432,635.8)	37.3 (23.3–57.7)	−0.063 (−0.162–0.035)
Low‐middle	256,609.8 (160,692.6–398,577.0)	35.0 (21.9–54.4)	261,278.5 (161,829.4–403,860.8)	34.2 (21.2–52.8)	−0.218 (−0.288‐0.148)
Low	113,625.6 (71,637.0–174,822.9)	24.2 (15.3–37.3)	140,851.7 (88,559.6–217,435.0)	24.1 (15.2–37.2)	−0.014 (−0.053–0.024)
Region					
Andean Latin America	10,465.2 (6513.9–16159.4)	47.7 (29.7–73.7)	11,088.9 (6877.4–17284.7)	46.8 (29.1–73.0)	−0.218 (−0.278‐0.158)
Australasia	3175.4 (1926.2–5037.7)	46.1 (28.0–73.2)	3372.9 (2056.3–5294.2)	44.7 (27.3–70.2)	−0.175 (−0.333‐0.017)
Caribbean	5397.7 (3273.9–8392.8)	34.6 (21.0–53.8)	5096.8 (3100.7–7896.7)	33.4 (20.3–51.7)	−0.409 (−0.495‐0.322)
Central Asia	9576.0 (5897.0–14905.8)	31.3 (19.3–48.7)	10,458.7 (6346.6–16257.7)	30.2 (18.3–47.0)	−0.297 (−0.399‐0.195)
Central Europe	5985.8 (3780.1–9424.5)	23.1 (14.6–36.4)	5491.5 (3402.9–8505.9)	23.3 (14.4–36.1)	0.068 (0.024–0.112)
Central Latin America	35,646.3 (21,809.9–54,886.8)	38.8 (23.8–59.8)	34,573.3 (21,189.1–53,204.2)	40.5 (24.8–62.4)	0.456 (0.251–0.662)
Central Sub‐Saharan Africa	8170.3 (4901.4–12,800.2)	14.6 (8.8–22.9)	11,516.7 (6983.0–18,322.1)	15.7 (9.5–24.9)	0.495 (0.379–0.611)
East Asia	90,412.4 (56,697.7–138,384.7)	27.0 (16.9–41.3)	97,695.7 (62,068.1–150,488.5)	28.3 (18.0–43.6)	0.808 (0.383–1.236)
Eastern Europe	16,523.4 (10,289.1–25,706.8)	36.9 (23.0–57.5)	17,531.9 (10,834.6–27,589.9)	38.0 (23.5–59.8)	0.303 (0.157–0.448)
Eastern Sub‐Saharan Africa	35,897.6 (22,865.0–54,293.5)	19.3 (12.3–29.2)	43,221.7 (27,505.7–66,309.8)	19.0 (12.1–29.1)	−0.133 (−0.187‐0.080)
High‐income Asia Pacific	13,105.3 (8137.4–20,241.0)	36.0 (22.4–55.7)	11,013.5 (6894.7–17,049.1)	35.8 (22.4–55.4)	−0.033 (−0.146–0.080)
High‐income North America	32,522.8 (20,314.1–50,382.1)	35.4 (22.1–54.9)	29,989.0 (18,816.5–46,454.8)	33.5 (21.0–51.9)	−0.593 (−0.682‐0.503)
The Middle East and North Africa	110,705.7 (68,611.3–168,253.5)	51.8 (32.1–78.7)	121,751.9 (75,234.7–186,518.7)	51.5 (31.8–78.9)	−0.052 (−0.158–0.055)
Oceania	1599.5 (949.7–2540.1)	31.3 (18.6–49.8)	2000.1 (1184.5–3176.6)	31.3 (18.5–49.7)	−0.017 (−0.050–0.015)
South Asia	225,756.6 (141,308.0–353,577.6)	33.1 (20.7–51.8)	222,012.3 (139,150.1–346,617.6)	32.5 (20.4–50.7)	−0.099 (−0.238–0.039)
Southeast Asia	96,949.4 (59,595.2–148,898.7)	41.8 (25.7–64.2)	94,809.9 (58,698.4–145,964.8)	41.4 (25.6–63.7)	−0.107 (−0.147‐0.068)
Southern Latin America	10,400.3 (6297.0–16,069.0)	51.5 (31.2–79.5)	10,038.3 (5932.2–15,703.4)	51.5 (30.4–80.5)	−0.026 (−0.172–0.119)
Southern Sub‐Saharan Africa	7096.8 (4436.2–11,146.9)	23.6 (14.7–37.0)	7460.3 (4689.4–11,638.5)	23.9 (15.0–37.2)	0.112 (0.087–0.136)
Tropical Latin America	46,701.2 (29,045.6–721,26.7)	67.1 (41.7–103.7)	39,575.3 (24,784.8–60,940.5)	59.4 (37.2–91.5)	−1.403 (−1.928‐‐0.875)
Western Europe	40,260.2 (24,894.8–62,674.8)	43.9 (27.2–68.4)	40,316.3 (24,888.5–62,666.0)	44.0 (27.1–68.3)	0.012 (−0.017–0.042)
Western Sub‐Saharan Africa	42,049.4 (26,793.6–63,963.6)	21.4 (13.7–32.6)	59,089.2 (37,159.5–90,352.5)	22.0 (13.8–33.6)	0.291 (0.167–0.414)

The burden was higher in females across most regions, except in Western Europe and high‐income Asia Pacific, where males had higher rates (Figure 2, Tables S1 and S2). In terms of age, 52.4% of the regions (11 regions) had the highest prevalence and YLD rates in the 15–19 age group in both 2010 and 2021 (Figure 3, Tables S3 and S4).

FIGURE 2Burden of refraction disorders by sex in global and 21 regions in 2010 and 2021. (a) Prevalent rates of burden of refraction disorders in 2010. (b) Prevalent rate of burden of refraction disorders in 2021. (c) YLD rates of burden of refraction disorders in 2010. (d) YLD rates of burden of refraction disorders in 2021. YLDs = years lived with disability.

**Figure:**
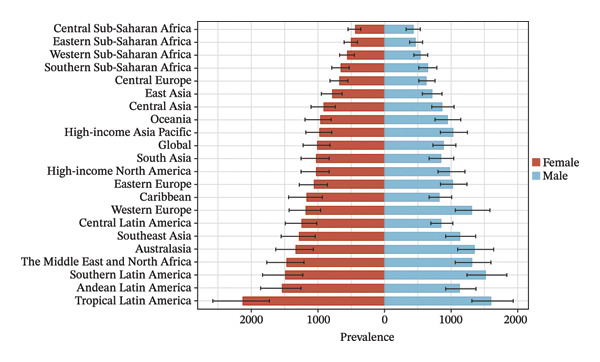
(a)

**Figure:**
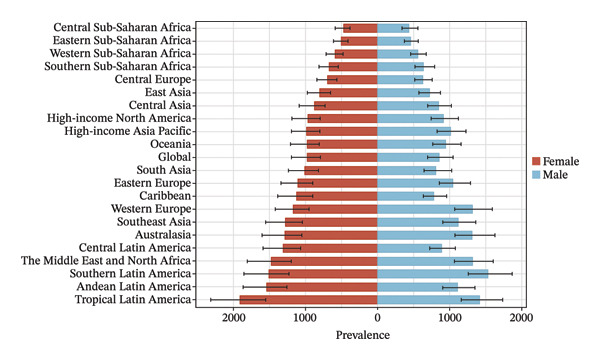
(b)

**Figure:**
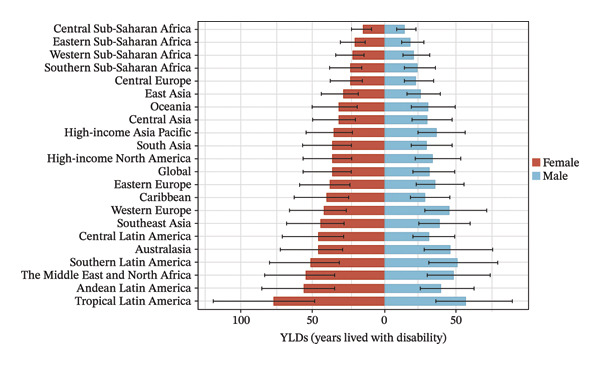
(c)

**Figure:**
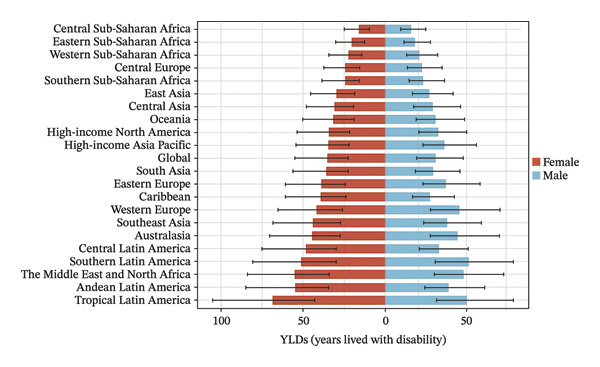
(d)

FIGURE 3Burden of refraction disorders by age in global and 21 regions in 2010 and 2021. (a) Prevalent rates of burden of refraction disorders in 2010 and 2021. (b) YLD rates of burden of refraction disorders in 2010 and 2021. YLDs = years lived with disability.

**Figure:**
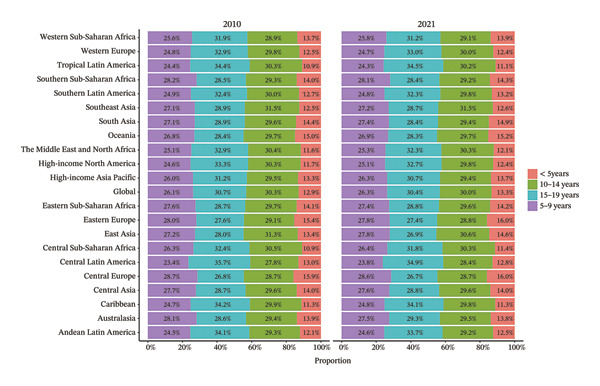
(a)

**Figure:**
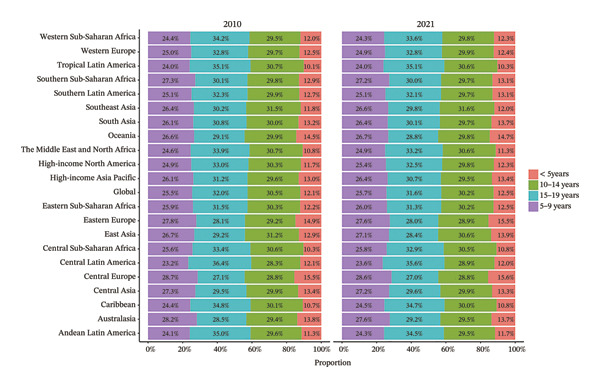
(b)

### 3.3. Burden Trends by Country

In 2010, the countries with the highest refraction disorders prevalence rates were Oman (2614.0 per 100,000), Spain (1983.0 per 100,000), and Saudi Arabia (1968.8 per 100,000), and in 2021, these three countries remained at the top. Regarding YLD rates, in 2010, Oman (91.2 per 100,000), Saudi Arabia (74.4 per 100,000), and Spain (72.1 per 100,000) had the highest rates. By 2021, Oman (87.4 per 100,000), Spain (75.0 per 100,000), and Saudi Arabia (72.9 per 100,000) still led in YLD rates. Overall, 40.7% of nations (83 countries) experienced an upward trend in both prevalence and YLD rates due to refraction disorders (Figure 4, Tables S5 and S6), with an EAPC greater than zero. The top three countries with the highest increases were Fiji, Mexico, and Nigeria. Conversely, 28.9% of countries (59 nations) saw a decline in prevalence and YLD rates (EAPC less than zero), with Brazil, the United States of America, and Singapore leading the list of countries with the most significant reductions.

FIGURE 4Global maps of prevalence rates (a) and years lived with disability (YLDs) (b) in 2021, as well as estimate annual percentage changes (EAPCs) in prevalence (c) and YLD rates (d) from 2010 to 2021. YLDs = years lived with disability.

**Figure:**
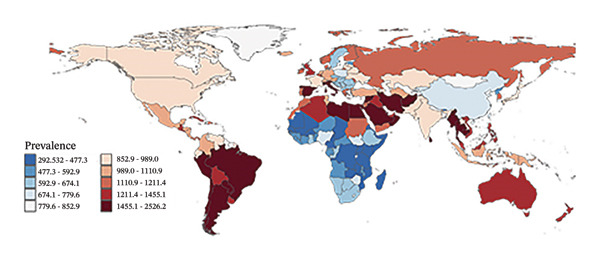
(a)

**Figure:**
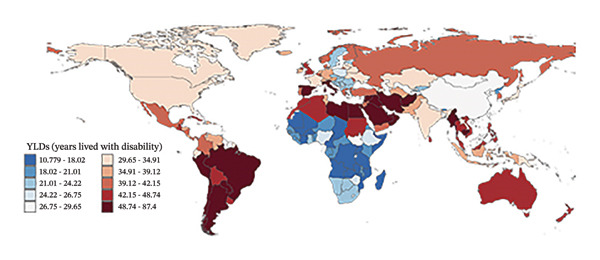
(b)

**Figure:**
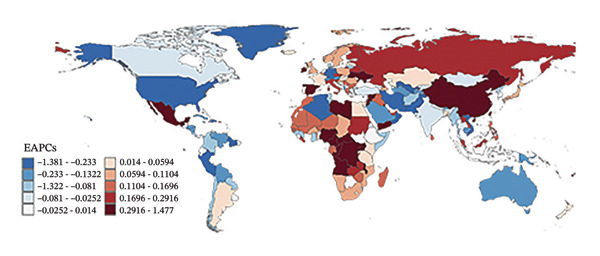
(c)

**Figure:**
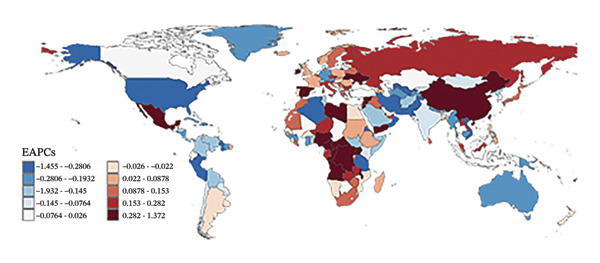
(d)

### 3.4. Burden Trends by Country SDI

We found that in 2021, the SDI was correlated with the prevalence rate and YLD rate caused by refractive disorders across 21 regions and 204 countries (Figure 5, Figure S1). However, there was no significant correlation between SDI and the EAPC of prevalence and YLD rates (Figure S2). Notably, from 2010 to 2021, the EAPC for prevalence rates showed a downward trend in both high SDI regions (−0.212, 95% UI: −0.288 to −0.148) and low‐middle SDI regions (−0.190, 95% UI: −0.242 to −0.137), while in middle‐high SDI regions, the EAPC indicated an upward trend (0.122, 95% UI: 0.008–0.237) (Table 1).

FIGURE 5Prevalent rates of burden of refraction disorders in 21 regions and 204 countries by SDI in 2021. SDI = sociodemographic index. (a) Prevalent rates of burden of refraction disorders in 21 regions by SDI in 2021. (b) Prevalent rate of burden of refraction disorders in 204 countries by SDI in 2021.

**Figure:**
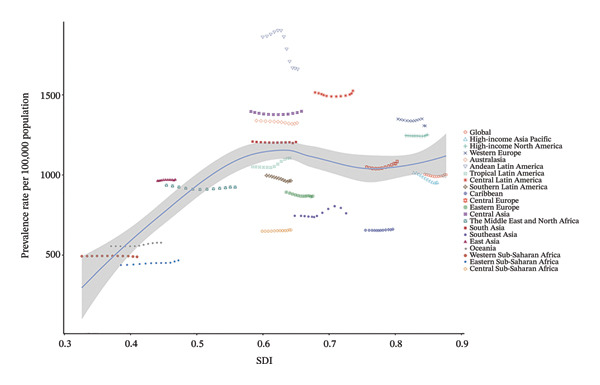
(a)

**Figure:**
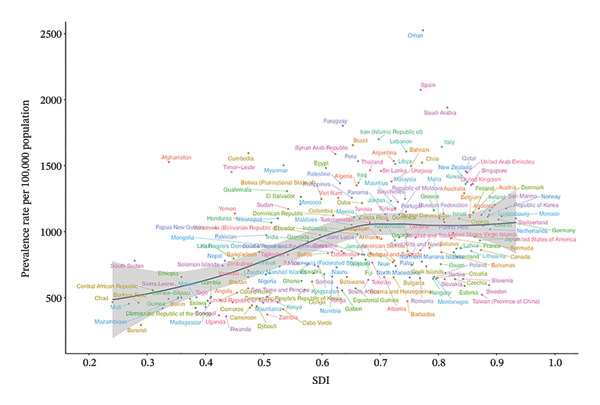
(b)

## 4. Discussion

Our analysis underscores trends in the global burden of refraction disorders, drawing attention to gender disparities and regional variations in healthcare resource allocation and service availability. It suggests that while overall improvements have been made in many regions, targeted interventions are crucial, particularly in middle‐high SDI areas, where the burden is increasing. This points to a gap in vision care and the need for public health efforts to address these issues, especially for females and older adolescents, who are disproportionately affected.

Compared to 2010, both the prevalence rate and YLD rate showed a declining trend in 2021. Our findings are consistent with Liu et al.’s analysis of GBD data from 1990 to 2019, which also demonstrated a decreasing trend in the burden of refraction disorders [5]. It is possible to link this decrease to global efforts, notably the “VISION 2020: The Right to Sight” initiative. Jointly launched by the WHO and IAPB in 1999, this campaign set the ambitious goal of eradicating avoidable blindness by 2020 [17]. The initiative highlighted that 7.26% of vision loss among individuals under 20 was due to refraction disorders, underscoring the importance of prioritizing eye health in children and adolescents [18]. In 2013, the WHO and IAPB introduced the “Universal Eye Health: A Global Action Plan 2014–2019,” aiming to reduce the prevalence of avoidable visual impairment by 25% by 2019 [19].

Our findings, derived from the GBD 2021 dataset, both align with and extend the insights from recent burden studies on refractive disorders. The overall declining trend in age‐standardized burden we observed among children and adolescents is consistent with the global trend reported for the all‐age population [7–9]. However, our age‐stratified analysis reveals critical nuances that refine the public health narrative. The significant decline in the 15–19 age group stands in contrast to studies noting an increasing number of cases in children and adolescents driven by population growth [10], suggesting that intensive, targeted interventions in late adolescence may be yielding measurable benefits. Conversely, the persistent and disproportionate burden on females aligns with the consistent sex disparity reported across recent GBD analyses [7, 8]. Most notably, our identification of an increasing burden in middle‐high SDI regions provides a crucial, focused confirmation and spatial delineation of the trend hinted at in studies reporting rising burdens in high SDI settings for younger populations [9, 10]. This moves the discussion from a general observation to a specific public health challenge for defined geographies. Therefore, while broader GBD 2021 studies establish the overall landscape and long‐term trends (e.g., 1990–2021), our analysis provides a complementary, detailed snapshot of the most recent decade with a dedicated focus on subpopulations, thereby highlighting the differential progress and emerging disparities that require tailored policy responses.

We found that in both 2010 and 2021, the global prevalence rate and YLD rate of refraction disorders were higher in females than in males. This gender disparity could be attributed to two main factors. First, socioeconomic and cultural barriers may limit women’s access to vision screening, correction, and treatment services. Second, biological differences could contribute to a higher risk of refraction disorders in females. The increased risk of myopia development in girls is partially explained by their earlier pubertal timing compared to their male counterparts [20, 21]. Fortunately, the EAPC for both prevalence and YLD rates during 2010–2021 was higher for females than males, suggesting increased attention to women’s eye health and the implementation of targeted interventions over the past decade [22, 23].

The most notable reduction in the prevalence and disability burden of refractive disorders occurred in the 15‐ to 19‐year‐old demographic [24]. The most notable reduction in burden among the 15–19 age group may be attributed not only to general awareness but also to the scaling up of targeted, evidence‐based interventions during the study period. For example, Singapore has implemented and evaluated comprehensive school‐based myopia control programs that combine elements such as mandatory annual vision screenings, provision of subsidized or free corrective eyewear, and the promotion of increased outdoor time through structured school activities [25]. Moreover, a study conducted on Chinese students indicates that engaging in outdoor activities during breaks or physical education classes, providing appropriate desks and chairs, and giving adequate guidance on reading and writing postures all contribute to preventing the pathological growth of the eyes [26]. These targeted efforts likely led to earlier detection, higher correction rates, and a consequent reduction in disability years in this age group.

In summary, from 2010 to 2021, the burden of refraction disorders increased in several regions. Tropical Latin America had the highest YLD rate in both 2010 (67.1 per 100,000 population) and 2021 (59.4 per 100,000 population). Despite the decline in the YLD rate, the burden of refraction disorders in this region remained significant. East Asia showed the largest increase in the EAPC of YLD rates, indicating a notable rise in the burden of refraction disorders in this region.

Over the past decade, lifestyle changes have occurred, particularly among children and adolescents, with outdoor activities being replaced by indoor activities and face‐to‐face learning shifting to online platforms [27, 28]. A cross‐sectional study from Hong Kong found that during the COVID‐19 pandemic, the shift to online learning and extended near‐work time led to a significant increase in myopia prevalence among children [29]. Notably, although the prevalence of refraction disorders significantly increased in East Asia by 2021 compared to 2010, the YLD rate did not change significantly. This could be linked to national eye health policies. For instance, in 2018, China introduced a joint initiative by eight ministries aimed at controlling myopia in children and adolescents, which is expected to reduce the prevalence of uncorrected refractive errors in the coming years [30].

During the period from 2010 to 2021, the burden of refraction disorders increased in most countries. Oman, Spain, and Saudi Arabia consistently exhibited the highest prevalence rates and YLDs associated with refractive errors, which may be attributed to two primary factors: first, environmental and lifestyle factors. In countries such as Spain and Saudi Arabia, the hot climate encourages residents to engage in indoor activities, thereby reducing outdoor activity time, which is considered a crucial measure for preventing myopia [31, 32]; second, government support. The Omani government has launched the “Vision 2050 in Oman” strategy aimed at comprehensively reducing the incidence of noncommunicable diseases (NCDs), resulting in more cases being diagnosed and treated [33]. In contrast, the countries with the most significant reduction in YLDs are Brazil, the United States, and Singapore. Brazil established the “Vision for the Future” project in 2009, which aims to prevent and treat visual impairments in children by providing vision screening, free medical assistance, and corrective devices, thereby striving to reduce the burden of uncorrected refractive errors and other vision problems. The United States and Singapore, as developed countries, possess some of the most advanced medical equipment and comprehensive healthcare systems globally [34]. Furthermore, the governments of these countries actively promote eye health education, emphasizing early prevention and intervention to maximize the protection of public eye health.

We found a positive correlation between the SDI and the prevalence and YLDs due to refractive disorders in 2021. This indicates that higher SDI levels are associated with higher prevalence rates and YLDs for refractive errors. From 2010 to 2021, the EAPC in the prevalence of refractive disorders showed a declining trend in high SDI regions and low‐middle SDI regions, while it exhibited an increasing trend in middle‐high SDI regions. The increasing trend observed in middle‐high SDI regions, despite their economic development, likely points to a lag in the development and accessibility of vision care services relative to socioeconomic growth. This hypothesis is supported by literature indicating disparities in access to eye care professionals (e.g., optometrists and ophthalmologists per capita) and inconsistent implementation of national school‐based vision screening programs in several such regions [35]. For instance, studies have shown that even within middle‐high SDI countries, coverage of effective refractive error correction (eREC) can be suboptimal, particularly in rural or under‐served urban areas, leading to underdiagnosis and undertreatment that may paradoxically drive up the measured burden as detection improves unevenly [36]. This underscores that economic advancement does not automatically translate to equitable eye health system maturity.

### 4.1. Limitations

This study has several limitations. First, estimates for regions with scarce epidemiological data (e.g., Central Sub‐Saharan Africa) rely on modeling and extrapolation, which may overestimate prevalence. Second, the GBD 2021 data lack information on risk factors, preventing causal inference. Finally, the absence of detailed subclassifications for refractive errors limits the analysis of specific conditions. Future GBD cycles should incorporate more granular data.

## 5. Conclusion

The prevalence and YLD rates of refractive disorders among children and adolescents declined overall from 2010 to 2021, with the most significant reduction in the 15–19 age group. However, females continue to bear a higher burden than males. Moreover, middle‐high SDI regions show an increasing burden, suggesting inadequate vision care resources and interventions. Future public health policies should prioritize female children and adolescents and middle‐high SDI regions by enhancing vision screening and interventions to reduce the global burden of refractive errors.

**Nomenclature**
GBD: Global Burden of Disease
WHO: World Health Organization
YLD: Years lived with disability
SDI: Sociodemographic index
MSVI: Moderate and serve visual acuity
VA: Visual acuity
UI: Uncertainty intervals

## Author Contributions

Jiayu Zhao and Xiao Guo designed the study, Jiayu Zhao wrote the main manuscript text and all Figures 1–5 and Supporting Figures 1 and 2. Jiayu Zhao and Lili Sun collected the data, conducted the analyses, and made Tables 1–2 and Supporting Figures 1–6. Xiao Guo and Lijun Zhang edited and revised the manuscript.

## Funding

This study was supported by the National Natural Science Foundation of China, Provincial Science and Technology Project 2022JH1/10800071 and 2023‐MSLH‐049.

## Disclosure

All authors have approved the submitted version and agreed with the contribution’s declarations.

## Ethics Statement

The authors have nothing to report, because Global Health Data Exchange (GHDx) belongs to public databases, the patients involved in the database have obtained ethical approval, users can download relevant data for free for research and publish relevant articles, and our study is based on open‐source data. The Affiliated Dalian Third People’s Hospital of Dalian Medical University does not require research using publicly available data to be submitted for review to their ethics committee, so there are no ethical issues and other conflicts of interest.

## Consent

The authors have nothing to report.

## Conflicts of Interest

The authors declare no conflicts of interest.

## Supporting Information

Additional supporting information can be found online in the Supporting Information section.

## Supporting information


**Supporting Information** Supporting Figure 1. YLD rates of burden of refraction disorders in 21 regions (A) and 204 countries (B) by SDI in 2021. SDI = sociodemographic index. Supporting Figure 2. EAPC of prevalence rates (A) and YLD rates (B) of refraction disorders burden in 204 countries by SDI from 2010 to 2021. Supporting Table 1. Prevalence rate of refraction disorders by sex in global and 21 regions in 2010 and 2021. Supporting Table 2. YLDs rate of refraction disorders by sex in global and 21 regions in 2010 and 2021. Supporting Table 3. Prevalence rate of refraction disorders by age in global and 21 regions in 2010 and 2021. Supporting Table 4. YLDs rate of refraction disorders by age in global and 21 regions in 2010 and 2021. Supporting Table 5. Prevalence rate of refractive disorders and average annual percentage changes from 2010 to 2021 at 204 nations. Supporting Table 6 Years lived with disability of refractive disorders and estimated annual percentage changes from 2010 to 2021 at 204 nations.

## Data Availability

The datasets used and/or analyzed during the current study were publicly available from the Global Health Data Exchange (https://ghdx.healthdata.org/gbd-results-tool).
