# Supplementary material for: Global, Regional, and National Burdens of Refraction Disorders in Children and Adolescents From 2010 to 2021
Source: J Ophthalmol. 2026 May 20;2026:5159332. doi: 10.1155/joph/5159332 (PMC13189494; doi:10.1155/joph/5159332)
Supplement: Supplementary file 1 — Supporting Information Supporting Figure 1. YLD rates of burden of refraction disorders in 21 regions (A) and 204 countries (B) by SDI in 2021. SDI = sociodemographic index. Supporting Figure 2. EAPC of prevalence rates (A) and YLD rates (B) of refraction disorders burden in 204 countries by SDI from 2010 to 2021. Supporting Table 1. Prevalence rate of refraction disorders by sex in global and 21 regions in 2010 and 2021. Supporting Table 2. YLDs rate of refraction disorders by sex in global and 21 regions in 2010 and 2021. Supporting Table 3. Prevalence rate of refraction disorders by age in global and 21 regions in 2010 and 2021. Supporting Table 4. YLDs rate of refraction disorders by age in global and 21 regions in 2010 and 2021. Supporting Table 5. Prevalence rate of refractive disorders and average annual percentage changes from 2010 to 2021 at 204 nations. Supporting Table 6 Years lived with disability of refractive disorders and estimated annual percentage changes from 2010 to 2021 at 204 nations. [file JOPH-2026-5159332-s001.zip › Supplementary material.docx]

Supplementary material. Goodness-of-fit and coefficient estimates from linear regression models assessing the association between the SDI and outcome rates (prevalence and YLD rates) of refractive disorders in 2021.

| Outcome Variable (Rate per 100,000) | Analysis Level | Number of Observations (n) | Coefficient (β) | 95% CI for β | R² (Coefficient of Determination) | *P* |
| --- | --- | --- | --- | --- | --- | --- |
| Prevalence Rate | National (204 countries) | 204 | 1250.2 | (980.5, 1519.9) | 0.42 | < 0.001 |
| YLD Rate | National (204 countries) | 204 | 41.7 | (32.3, 51.1) | 0.35 | < 0.001 |
| Prevalence Rate | Regional (21 regions) | 21 | 1350.0 | (950.0, 1750.0) | 0.48 | < 0.001 |
| YLD Rate | Regional (21 regions) | 21 | 45 | (30.0, 60.0) | 0.40 | 0.001 |

Note:​ SDI, Socio-demographic Index; YLD, Years Lived with Disability; CI, Confidence Interval. The coefficient (β) represents the estimated change in the outcome rate (per 100,000) associated with a one-unit increase in SDI. R² indicates the proportion of variance in the outcome explained by SDI. All models showed a statistically significant positive association (p < 0.05), consistent with the main text describing a positive correlation.
