# Supplementary material for: Global, Regional, and National Burdens of Refraction Disorders in Children and Adolescents From 2010 to 2021
Source: J Ophthalmol. 2026 May 20;2026:5159332. doi: 10.1155/joph/5159332 (PMC13189494; doi:10.1155/joph/5159332)
Supplement: Supplementary file 1 — Supporting Information Supporting Figure 1. YLD rates of burden of refraction disorders in 21 regions (A) and 204 countries (B) by SDI in 2021. SDI = sociodemographic index. Supporting Figure 2. EAPC of prevalence rates (A) and YLD rates (B) of refraction disorders burden in 204 countries by SDI from 2010 to 2021. Supporting Table 1. Prevalence rate of refraction disorders by sex in global and 21 regions in 2010 and 2021. Supporting Table 2. YLDs rate of refraction disorders by sex in global and 21 regions in 2010 and 2021. Supporting Table 3. Prevalence rate of refraction disorders by age in global and 21 regions in 2010 and 2021. Supporting Table 4. YLDs rate of refraction disorders by age in global and 21 regions in 2010 and 2021. Supporting Table 5. Prevalence rate of refractive disorders and average annual percentage changes from 2010 to 2021 at 204 nations. Supporting Table 6 Years lived with disability of refractive disorders and estimated annual percentage changes from 2010 to 2021 at 204 nations. [file JOPH-2026-5159332-s001.zip › Supplementary Table 2.docx]

Supplementary Table 2.YLDs Rate Refraction Disorders by sex in global and 21 regions in 2010 and 2021.

|  | YLDs Rate | | | |
| --- | --- | --- | --- | --- |
|  | 2010, (per 100 000 population) | | 2021, (per 100 000 population) | |
|  | Female | Male | Female | Male |
| Global | 36.7  (22.9-56.5) | 31.8  (19.8-49.0) | 35.7  (22.3-55.1) | 31.1  (19.3-48.2) |
| Region |  |  |  |  |
| Andean Latin  America | 56.0  (34.5-85.4) | 39.7  (24.5-62.5) | 55.0  （34.3-84.8） | 39.2  （24.2-61.7） |
| Australasia | 46.3  (28.7-72.4) | 46.0  (27.6-75.4) | 44.7  （27.1-70.1） | 44.8  （27.4-70.7） |
| Caribbean | 40.5  (24.6-62.7) | 28.9  (17.6-45.3) | 39.4  （24.1-60.8） | 27.6  （16.6-42.7） |
| Central Asia | 912.3  (744.9-1098.2) | 873.3  (706.4-1057.2) | 31.0  （19.1-48.2） | 29.5  （17.8-46.5） |
| Central Europe | 24.2  (15.1-37.8) | 22.0  (13.7-34.5) | 39.1  （24.2-60.8） | 36.9  （22.7-58.4） |
| Central Latin  America | 46.1  (28.1-70.9) | 31.7  (19.5-49.1) | 48.4  （29.7-74.8） | 32.9  （20.4-51.2） |
| Central Sub Saharan  Africa | 15.0  (8.9-23.5) | 14.3  (8.3-22.4) | 16.0  （9.7-25.2） | 15.3  （9.131743-24.49833） |
| East Asia | 28.7  (18.0-44.0) | 25.5  (15.9-39.1) | 29.6  （18.5-45.7） | 27.2  （17.5-41.8） |
| Eastern Europe | 38.1  (23.7-59.5) | 35.8  (22.4-55.6) | 39.1  （24.2-60.8） | 36.9  （22.7-58.4） |
| Eastern SubeSaharan  Africa | 20.3  (12.9-30.8) | 18.3  (11.7-27.7) | 19.9  （12.6-30.2） | 18.1  （11.5-27.8） |
| High-income  Asia Pacifc | 57.6  (39.3-84.5) | 60.3  (41.9-86.8) | 35.1  （21.8-54.5） | 36.4  （22.7-56.3） |
| High-income North  America | 42.4  (28.6-63.2) | 47.1  (32.2-69.4) | 34.5  （21.5-53.6） | 32.5  （20.5-50.1） |
| North Africa and  Middle East | 86.2  (58.0-127.3) | 97.9  (66.8-144.0) | 54.8  （33.9-84.0） | 48.4  （30.2-73.7） |
| Oceania | 32.0  (18.9-50.4) | 30.7  (18.3-49.2) | 32.0  （19.0-50.4） | 30.7  （18.6-49.0） |
| South Asia | 36.3  (22.9-56.9) | 30.1  (18.7-47.1) | 36.0  （22.7-56.3） | 29.2  （18.0-46.1） |
| Southeast Asia | 44.7  (27.8-68.2) | 39.1  (23.7-60.3) | 44. 2  （27.2-68.4） | 38.7  （24.1-59.6） |
| Southern Latin  America | 51.6  (31.2-80.0) | 51.3  (30.9-79.3) | 51.3  （29.8-80.8） | 51.6  （31.0-79.6） |
| Southern  SubeSaharan Africa | 24.2  (15.3-37.9) | 23.0  (14.3-35.9) | 24.6  （15.6-38.6） | 23.1  （14.4-36.3） |
| Tropical Latin  America | 57.3  (35.6-89.4) | 77.3  (48.3-119.3) | 68.5  （43.0-105.4） | 50.7  （31.5-79.2） |
| Western Europe | 42.3  (26.2-65.6) | 45.4  (28.0-71.6) | 42.0  （26.0-65.2） | 45.9  （28.3-71.5） |
| Western  SubeSaharan Africa | 22.3  (14.1-34.0) | 20.7  (13.1-31.4) | 22.7  （14.3-34.7） | 21.3  （13.3-32.4） |
