# Supplementary material for: Global, Regional, and National Burdens of Refraction Disorders in Children and Adolescents From 2010 to 2021
Source: J Ophthalmol. 2026 May 20;2026:5159332. doi: 10.1155/joph/5159332 (PMC13189494; doi:10.1155/joph/5159332)
Supplement: Supplementary file 1 — Supporting Information Supporting Figure 1. YLD rates of burden of refraction disorders in 21 regions (A) and 204 countries (B) by SDI in 2021. SDI = sociodemographic index. Supporting Figure 2. EAPC of prevalence rates (A) and YLD rates (B) of refraction disorders burden in 204 countries by SDI from 2010 to 2021. Supporting Table 1. Prevalence rate of refraction disorders by sex in global and 21 regions in 2010 and 2021. Supporting Table 2. YLDs rate of refraction disorders by sex in global and 21 regions in 2010 and 2021. Supporting Table 3. Prevalence rate of refraction disorders by age in global and 21 regions in 2010 and 2021. Supporting Table 4. YLDs rate of refraction disorders by age in global and 21 regions in 2010 and 2021. Supporting Table 5. Prevalence rate of refractive disorders and average annual percentage changes from 2010 to 2021 at 204 nations. Supporting Table 6 Years lived with disability of refractive disorders and estimated annual percentage changes from 2010 to 2021 at 204 nations. [file JOPH-2026-5159332-s001.zip › Supplementary Table 5.docx]

Supplementary Table 5. Prevalence Rate of Refractive Disorders and Average Annual Percentage Changes from 2010 to 2021 at 204 nations

|  | Prevalence Rate | | | | |
| --- | --- | --- | --- | --- | --- |
|  | Case(n), 2010 | Prevalence, 2010 (per 100 000 population) | Case(n), 2021 | Prevalence, 2010 (per 100 000 population) | EAPC  2010-2021 |
| Nation |  |  |  |  |  |
| Afghanistan | 205823.9  (161305.2-255624.9) | 1542.9  (1209.2-1916.2) | 271413.4  (215832.8-337999.1) | 1526.8  (1214.1-1901.4) | -0.090  (-0.121--0.059) |
| Albania | 6053.0  (4869.2-7324.3) | 666.7  (536.3-806.7) | 4048.4  (3253.3-4897.1) | 656.0  (527.2-793.5) | -0.145  (-0.219--0.071) |
| Algeria | 190532.4  (153903.9-230302.2) | 1386.5  (1120.0-1675.9) | 222235.2  (179412.8-271027.6) | 1351.1  (1090.7-1647.7) | -0.245  (-0.469--0.020) |
| American Samoa | 201.1  (159.1-245.1) | 763.9  (604.3-931.3) | 149.2  (118.4-185.2) | 780.0  (618.9-968.0) | 0.167  (0.130-0.204) |
| Andorra | 181.7  (145.5-220.7) | 1127.6  (903.2-1369.5) | 169.1  (136.8-206.9) | 1175.8  (951.2-1438.8) | 0.440  (0.392-0.487) |
| Angola | 64241.7  (50107.6-79565.8) | 515.5  (402.1-638.4) | 100365.5  (78085.7-123659.7 | 537.4  (418.1-662.1) | 0.301  (0.187-0.414) |
| Antigua and Barbuda | 234.0  (187.1-287.8) | 833.7  (666.7-1025.6) | 191.1  (150.4-234.9) | 818.0  (643.8-1005.4) | -0.234  (-0.312--0.156) |
| Argentina | 214531.3  (173758.8-259848.8) | 1517.4  (1229.0-1837.9) | 210497.5  (169041.9-256168.0) | 1535.2  (1232.8-1868.3) | -0.234  (-0.312--0.156) |
| Armenia | 8079.6  (6559.1-9778.6) | 950.3  (771.4-1150.1) | 7213.5  (5846.0-8790.8) | 945.3  (766.1-1152.0) | -0.003  (-0.156-0.150) |
| Australia | 75995.4  (60978.2-92389.4) | 1342.7  (1077.4-1632.4) | 80573.1  (65397.9-100305.4) | 1291.6  (1048.3-1607.9) | -0.229  (-0.438--0.019) |
| Austria | 19643.8  (15702.6-23832.0) | 1133.2  (905.8-1374.8) | 19490.3  (15853.4-23598.8) | 1110.6  (903.4-1344.7) | -0.218  (-0.269--0.168) |
| Azerbaijan | 29748.8  (24315.7-36236.9) | 957.1  (782.3-1165.9) | 29206.0  (23704.1-35782.8) | 958.6  (778.0-1174.4) | 0.061  (-0.062-0.184) |
| Bahamas | 1012.6  (808.7-1235.2) | 823.0  (657.3-1003.9) | 972.9  (767.7-1198.6) | 844.0  (666.1-1039.9) | 0.254  (0.211-0.297) |
| Bahrain | 4744.6  (3848.7-5744.7) | 1458.9  (1183.4-1766.4) | 6041.6  (4892.0-7382.3) | 1497.2  (1212.3-1829.5) | 0.286  (0.228-0.343) |
| Bangladesh | 555326.9  (438557.0-685218.8) | 843.1  (665.8-1040.3) | 515716.7  (403264.3-638477.2) | 845.6  (661.2-1046.9) | 0.025  (-0.000-0.051) |
| Barbados | 351.0  (284.2-428.1) | 465.0  (376.6-567.2) | 313.9  (251.9-384.7) | 472.9  (379.4-579.6) | 0.109  (0.061-0.157) |
| Belarus | 17781.5  (14448.8-21446.5) | 866.6  (704.2-1045.3) | 17780.9  (14412.7-21565.0) | 878.0  (711.6-1064.8) | 0.173  (-0.020-0.367) |
| Belgium | 27816.8  (22809.0-34245.8) | 1117.0  (915.9-1375.1) | 28660.2  (23355.8-34517.6) | 1126.5  (918.0-1356.7) | 0.087  (0.008-0.166) |
| Belize | 1261.3  (1010.5-1543.9) | 826.6  (662.3-1011.9) | 1414.4  (1122.3-1744.2) | 836.7  (663.9-1031.8) | 0.077  (0.048-0.105) |
| Benin | 26582.8  (21454.5-32455.4) | 490.6  (395.9-598.9) | 36815.8  (29436.1-44452.4) | 489.8  (391.6-591.4) | -0.049  (-0.096--0.002) |
| Bermuda | 134.6  (106.4-164.4) | 939.5  (742.4-1146.9) | 109.1  (88.2-133.6) | 949.0  (767.5-1161.7) | 0.053  (-0.017-0.123) |
| Bhutan | 1681.2  (1359.3-2041.5) | 577.8  (467.2-701.6) | 1454.1  (1174.1-1793.0) | 570.3  (460.5-703.2) | -0.169  (-0.212--0.126) |
| Bolivia (Plurinational State of) | 55971.0  (45284.2-67315.1) | 1258.5  (1018.2-1513.6) | 56710.2  (45519.8-69042.8) | 1247.3  (1001.1-1518.5) | -0.145  (-0.214--0.076) |
| Bosnia and Herzegovina | 5726.8  (4633.0-6956.0) | 656.5  (531.1-797.4) | 4347.1  (3500.3-5344.5) | 653.6  (526.3-803.6) | -0.027  (-0.110-0.055) |
| Botswana | 5794.4  (4533.1-7166.0) | 657.5  (514.3-813.1) | 6036.1  (4687.2-7551.5) | 658.2  (511.1-823.5) | 0.011  (-0.042-0.064) |
| Brazil | 1246898.1  (1018009.9-1505219.4) | 1863.5  (1521.4-2249.6) | 1058051.0  (859583.6-1276822.3) | 1655.4  (1344.8-1997.6) | -1.381  (-1.968--0.790) |
| Brunei Darussalam | 1493.3  (1196.1-1849.3) | 1082.3  (866.9-1340.4) | 1383.5  (1120.8-1683.1) | 1071.4  (867.9-1303.4) | -0.092  (-0.112--0.073) |
| Bulgaria | 8848.4  (7140.6-10802.6) | 638.2  (515.1-779.2) | 8408.1  (6825.3-10270.3) | 649.6  (527.3-793.5) | 0.169  (0.112-0.225) |
| Burkina Faso | 44330.2  (35666.2-54055.0) | 456.8  (367.5-557.0) | 59155.0  (46989.9-71573.9) | 461.9  (366.9-558.9) | 0.099  (0.055-0.144) |
| Burundi | 15233.1  (12259.4-18597.7) | 288.8  (232.4-352.6) | 21280.4  (16991.0-26035.4) | 292.5  (233.6-357.9) | 0.162  (0.123-0.202) |
| Cabo Verde | 934.5  (762.1-1130.1) | 415.3  (338.7-502.2) | 794.2  (642.6-960.5) | 412.1  (333.4-498.3) | -0.087  (-0.131--0.044) |
| Cambodia | 105417.3  (85965.3-128494.3) | 1618.7  (1320.0-1973.0) | 105746.4  (85069.6-129864.0) | 1594.7  (1282.9-1958.4) | -0.144  (-0.184--0.103) |
| Cameroon | 51299.0  (42254.3-62576.5) | 422.4  (347.9-515.2) | 74724.4  (61068.4-91137.7) | 442.1  (361.3-539.2) | 0.506  (0.351-0.661) |
| Canada | 68828.0  (55583.6-84137.0) | 865.1  (698.7-1057.6) | 70817.2  (57277.7-85624.6) | 860.6  (696.1-1040.5) | -0.072  (-0.122--0.021) |
| Central African Republic | 13500.7  (10474.0-16575.6) | 537.8  (417.2-660.3) | 16167.4  (12750.9-19804.9) | 558.7  (440.6-684.3) | 0.293  (0.197-0.389) |
| Chad | 33391.6  (26987.3-40422.1) | 461.3  (372.8-558.4) | 50986.5  (40733.6-62832.4) | 465.6  (372.0-573.8) | 0.094  (0.081-0.107) |
| Chile | 77699.7  (63244.5-93913.7) | 1535.6  (1249.9-1856.0) | 74536.8  (60596.4-90811.4) | 1523.1  (1238.2-1855.7) | -0.081  (-0.208-0.047) |
| China | 2424563.1  (1962449.6-2939538.0) | 753.5  (609.8-913.5) | 2566847.9  (2074665.9-3129976.4) | 767.8  (620.6-936.3) | 0.617  (0.188-1.048) |
| Colombia | 185576.2  (150208.1-223195.3) | 1119.1  (905.8-1345.9) | 161489.5  (132376.6-193317.3) | 1109.2  (909.2-1327.8) | -0.118  (-0.190--0.045) |
| Comoros | 1325.2  (1030.5-1644.5) | 429.9  (334.3-533.5) | 1332.4  (1038.8-1682.3) | 424.4  (330.9-535.8) | -0.091  (-0.136--0.045) |
| Congo | 11968.2  (9143.5-14883.1) | 578.7  (442.1-719.7) | 15479.4  (12024.5-19246.2) | 622.5  (483.5-773.9) | 0.617  (0.456-0.779) |
| Cook Islands | 46.7  (37.1-57.4) | 737.7  (585.3-905.8) | 38.2  (30.2-46.1) | 736.8  (582.4-890.0) | -0.006  (-0.080-0.068) |
| Costa Rica | 15715.0  (12900.3-18742.5) | 1017.6  (835.4-1213.7) | 13816.2  (11200.1-16545.2) | 1009.2  (818.1-1208.6) | -0.129  (-0.187--0.071) |
| Croatia | 6010.7  (4829.5-7339.7) | 646.0(519.0-788.8) | 5241.4  (4235.6-6453.1) | 650.5  (525.7-800.9) | 0.085  (0.043-0.126) |
| Cuba | 34539.3  (27793.2-41453.9) | 1241.2  (998.8-1489.7) | 29218.6  (23406.1-35370.9) | 1216.8  (974.8-1473.1) | -0.214  (-0.291--0.137) |
| Cyprus | 3086.5  (2522.5-3785.2) | 1136.0  (928.4-1393.2) | 3132.3  (2527.3-3809.8) | 1098.7  (886.5-1336.4) | -0.351  (-0.442--0.261) |
| Czechia | 13099.7  (10700.5-15873.0) | 626.3  (511.6-758.9) | 14226.8  (11441.1-17622.4) | 640.7  (515.2-793.6) | 0.201  (0.179-0.223) |
| Côte d'Ivoire | 53684.7  (41968.3-65564.2) | 480.2  (375.4-586.5) | 69293.5  (54779.7-84512.1) | 481.7  (380.8-587.5) | 0.026  (-0.017-0.070) |
| Democratic People's Republic of Korea | 44690.1  (35666.4-54524.2) | 563.5  (449.7-687.5) | 36936.4  (29476.2-45791.9) | 559.4  (446.5-693.6) | -0.086  (-0.135--0.037) |
| Democratic Republic of the Congo | 147134.5  (113597.5-184025.5) | 393.1  (303.5-491.7) | 202452.5  (156741.1-251736.8) | 422.6  (327.2-525.5) | 0.548  (0.428-0.669) |
| Denmark | 16424.8  (13384.5-19829.9) | 1215.8  (990.8-1467.9) | 15595.5  (12627.1-18953.0) | 1205.0  (975.7-1464.4) | -0.111  (-0.187--0.035) |
| Djibouti | 1765.9  (1411.6-2154.4) | 439.4  (351.2-536.0) | 2299.3  (1804.6-2803.6) | 435.2  (341.5-530.6) | -0.044  (-0.098-0.010) |
| Dominica | 198.9  (156.8-243.3) | 835.3  (658.7-1021.9) | 165.4  (131.4-201.6) | 851.4  (676.6-1038.0) | 0.170  (0.058-0.281) |
| Dominican Republic | 45610.5  (36341.8-56277.3) | 1165.4  (928.5-1437.9) | 43680.6  (34842.4-52912.5) | 1122.7  (895.5-1360.0) | -0.425  (-0.492--0.359) |
| Ecuador | 61672.7  (49814.8-74622.5) | 978.4  (790.3-1183.8) | 64881.5  (52487.6-78931.1) | 980.6  (793.3-1193.0) | -0.001  (-0.031-0.029) |
| Egypt | 551929.1  (451702.2-658908.5) | 1483.2  (1213.9-1770.7) | 690052.7  (555054.6-852326.5) | 1482.4  (1192.4-1831.0) | 0.030  (-0.165-0.224) |
| El Salvador | 34380.1  (28151.6-41161.5) | 1295.6  (1060.9-1551.1) | 30086.4  (24473.3-36166.5) | 1263.0  (1027.4-1518.3) | -0.312  (-0.381--0.243) |
| Equatorial Guinea | 3260.9  (2570.6-4006.3) | 556.8  (438.9-684.1) | 4390.6  (3398.8-5457.9) | 570.1  (441.3-708.7) | 0.168  (0.042-0.295) |
| Eritrea | 13543.3  (10596.6-16743.7) | 482.6  (377.6-596.6) | 15352.8  (11960.4-19065.3) | 477.3  (371.8-592.7) | -0.067  (-0.096--0.038) |
| Estonia | 1625.6  (1331.7-1973.6) | 578.3  (473.8-702.1) | 1651.5  (1342.6-2012.3) | 589.2  (479.0-717.9) | 0.190  (0.171-0.209) |
| Eswatini | 3805.6  (3054.1-4595.5) | 682.5  (547.8-824.2) | 3666.9  (2973.5-4436.4) | 685.4  (555.8-829.2) | 0.007  (-0.058-0.073) |
| Ethiopia | 325239.4  (260923.4-398028.8) | 661.4  (530.6-809.4) | 375850.5  (302871.9-455471.2) | 657.1  (529.5-796.3) | -0.006  (-0.138-0.125) |
| Fiji | 2314.9  (1861.3-2788.0) | 692.8  (557.0-834.3) | 2761.7  (2207.7-3394.6) | 787.7  (629.6-968.1) | 1.477  (0.989-1.967) |
| Finland | 13721.0  (11273.2-16652.8) | 1125.0  (924.3-1365.4) | 13164.4  (10589.1-16179.3) | 1146.2  (922.0-1408.7) | 0.202  (0.097-0.307) |
| France | 136593.4  (110697.9-166436.3) | 866.6  (702.3-1055.9) | 137750.5  (110822.6-167969.7) | 874.5  (703.6-1066.4) | 0.024  (-0.137-0.186) |
| Gabon | 4009.4  (3159.4-4967.3) | 541.5  (426.7-670.8) | 4588.9  (3591.8-5741.5) | 554.8  (434.3-694.2) | 0.164  (0.060-0.268) |
| Gambia | 4829.7  (3757.9-5968.5) | 492.0  (382.8-608.0) | 6408.0  (5007.2-7878.0) | 505.9  (395.3-622.0) | 0.259  (0.238-0.279) |
| Georgia | 9764.8  (7975.0-11690.8) | 962.1  (785.8-1151.9) | 8901.8  (7245.2-10822.5) | 957.9  (779.6-1164.6) | -0.041  (-0.173-0.090) |
| Germany | 169885.3  (136222.9-207580.1) | 1121.0  (898.9-1369.7) | 174166.3  (140504.9-212466.4) | 1092.2  (881.1-1332.3) | -0.274  (-0.307--0.241) |
| Ghana | 80872.3  (64276.8-97133.5) | 633.7  (503.7-761.2) | 102258.4  (82036.7-124153.8) | 626.8  (502.9-761.0) | -0.110  (-0.146--0.074) |
| Greece | 25302.7  (20213.9-31042.6) | 1145.1  (914.8-1404.9) | 22141.6  (17633.9-27198.4) | 1162.1  (925.5-1427.5) | 0.143  (0.123-0.163) |
| Greenland | 150.2  (122.7-182.1) | 862.5  (704.7-1045.7) | 129.6  (104.9-155.5) | 843.4  (682.6-1011.7) | -0.285  (-0.368--0.202) |
| Grenada | 311.1  (245.8-379.9) | 249.0  (198.3-306.6) | 832.0  (657.3-1015.8) | 827.3  (658.8-1018.5) | -0.131  (-0.205--0.057) |
| Guam | 456.2  (363.4-555.4) | 761.4  (606.5-926.9) | 365.3  (290.2-444.9) | 753.3  (598.5-917.5) | -0.133  (-0.201--0.065) |
| Guatemala | 90476.7  (73481.7-109468.8) | 1305.0  (1059.9-1578.9) | 86488.6  (70792.7-103371.9) | 1307.5  (1070.2-1562.7) | -0.003  (-0.030-0.024) |
| Guinea | 27703.2  (22074.2-33618.2) | 494.7  (394.2-600.4) | 36636.1  (29077.2-45228.8) | 490.8  (389.5-605.9) | -0.088  (-0.192-0.016) |
| Guinea-Bissau | 4332.2  (3419.9-5314.3) | 493.9  (389.9-605.9) | 5611.1  (4437.0-6805.3) | 501.7  (396.7-608.4) | 0.129  (0.011-0.247) |
| Guyana | 2680.4  (2119.4-3314.0) | 833.8  (659.3-1030.9) | 2240.2  (1779.0-2802.0) | 798.0  (633.7-998.2) | -0.504  (-0.582--0.427) |
| Haiti | 38930.8  (31084.6-48305.1) | 808.7  (645.7-1003.5) | 44646.9  (35297.5-54931.1) | 796.2  (629.4-979.6) | -0.208  (-0.300--0.116) |
| Honduras | 42683.2  (34552.9-51640.1) | 1082.5  (876.3-1309.7) | 46494.8  (37432.3-56152.0) | 1068.7  (860.4-1290.7) | -0.142  (-0.187--0.097) |
| Hungary | 13331.8  (10663.2-16376.7) | 641.3  (512.9-787.8) | 12079.6  (9700.5-14661.4) | 644.1  (517.3-781.8) | 0.015  (-0.038-0.067) |
| Iceland | 944.0  (755.0-1154.5) | 1048.5  (838.6-1282.2) | 931.7  (747.2-1161.5) | 1045.6  (838.6-1303.5) | 0.050  (-0.019-0.120) |
| India | 4820359.0  (3814889.5-5940102.5) | 937.3  (741.8-1155.1) | 4638509.5  (3642256.5-5687902.5) | 926.6  (727.6-1136.3) | -0.046  (-0.240-0.149) |
| Indonesia | 923239.2  (736471.3-1125942.5) | 1010.0  (805.7-1231.7) | 911985.4  (728870.3-1116573.0) | 1010.9  (807.9-1237.6) | -0.007  (-0.048-0.033) |
| Iran (Islamic Republic of) | 439697.2  (358013.6-527885.3) | 1745.4  (1421.1-2095.4) | 439596.9  (357052.9-536056.3) | 1700.3  (1381.0-2073.4) | -0.339  (-0.486--0.191) |
| Iraq | 206359.4  (165964.8-253438.8) | 1342.0  (1079.3-1648.2) | 241718.5  (189988.6-298680.1) | 1370.5  (1077.2-1693.5) | 0.165  (0.081-0.249) |
| Ireland | 13788.3  (11291.8-16789.5) | 1087.7  (890.8-1324.5) | 14897.9  (11999.3-18110.0) | 1127.3  (907.9-1370.3) | 0.352  (0.297-0.406) |
| Israel | 30584.6  (24551.8-37134.9) | 1087.8  (873.3-1320.8) | 36909.2  (30075.2-45104.7) | 1094.1  (891.5-1337.1) | 0.036  (-0.027-0.100) |
| Italy | 182054.3  (148247.3-219805.4) | 1591.8  (1296.2-1921.9) | 172124.0  (139196.1-208186.7) | 1642.  5(1328.3-1986.6) | 0.266  (0.218-0.313) |
| Jamaica | 8572.4  (6784.7-10606.3) | 842.7  (667.0-1042.7) | 6813.6  (5426.8-8404.1) | 835.6  (665.6-1030.7) | -0.165  (-0.245--0.085) |
| Japan | 217962.2  (177387.9-263723.8) | 923.2  (751.3-1117.0) | 198262.7  (160147.2-240165.8) | 933.7  (754.2-1131.1) | 0.109  (0.039-0.180) |
| Jordan | 39073.3  (30964.7-47669.6) | 1119.5  (887.2-1365.8) | 58056.4  (45605.9-72164.7) | 1178.8  (926.0-1465.2) | 0.522  (0.416-0.628) |
| Kazakhstan | 50643.6  (42023.1-60604.6) | 935.4  (776.2-1119.3) | 62737.6  (50841.7-76270.5) | 933.0  (756.1-1134.3) | 0.026  (-0.061-0.112) |
| Kenya | 102119.5  (81732.1-124030.5) | 462.7  (370.3-562.0) | 114397.2  (91273.5-139767.1) | 465.0  (371.0-568.1) | 0.059  (0.029-0.089) |
| Kiribati | 362.6  (289.5-439.6) | 736.2  (587.7-892.4) | 393.3  (313.8-483.5) | 735.9  (587.2-904.7) | 0.008  (-0.032-0.048) |
| Kuwait | 11280.3  (9125.2-13678.0) | 1305.8  (1056.3-1583.3) | 14909.6  (12066.9-18226.1) | 1361.0  (1101.5-1663.8) | 0.376  (0.283-0.469) |
| Kyrgyzstan | 16102.6  (12981.7-19878.9) | 683.0  (550.6-843.2) | 19134.9  (15263.4-23213.2) | 677.9  (540.7-822.3) | -0.026  (-0.157-0.106) |
| Lao People's Democratic Republic | 26011.6  (20843.9-31675.2) | 857.0  (686.8-1043.7) | 26487.5  (21497.7-32332.9) | 885.4  (718.6-1080.8) | 0.212  (-0.120-0.545) |
| Latvia | 3723.6  (3025.0-4523.8) | 866.4  (703.9-1052.6) | 3374.5  (2701.8-4123.1) | 871.8  (698.0-1065.2) | 0.037  (-0.094-0.168) |
| Lebanon | 23374.1  (19175.2-28426.5) | 1601.9  (1314.2-1948.2) | 26768.6  (21846.3-32470.5) | 1606.5  (1311.1-1948.7) | -0.039  (-0.248-0.171) |
| Lesotho | 6046.7  (4792.7-7368.5) | 682.6  (541.1-831.9) | 5744.9  (4608.0-7008.1) | 684.9  (549.3-835.5) | 0.024  (-0.013-0.062) |
| Liberia | 10534.0  (8349.1-12820.4) | 482.5  (382.4-587.2) | 13762.8  (10937.6-16842.8) | 493.3  (392.0-603.7) | 0.181(0.158-0.204) |
| Libya | 34697.8  (27719.8-42865.7) | 1467.7  (1172.5-1813.2) | 31603.5  (25027.4-38898.2) | 1513.9  (1198.9-1863.4) | 0.323  (0.206-0.440) |
| Lithuania | 6013.2  (4814.3-7369.8) | 861.7  (689.9-1056.1) | 4570.7  (3641.0-5560.3) | 853.1  (679.6-1037.8) | -0.107  (-0.178--0.037) |
| Luxembourg | 1336.0  (1078.8-1632.7) | 1122.8  (906.6-1372.1) | 1502.3  (1215.3-1822.2) | 1113.5  (900.8-1350.6) | -0.082  (-0.108--0.055) |
| Madagascar | 50502.5  (39137.8-63133.6) | 421.5  (326.7-526.9) | 64084.2  (50648.7-79110.8) | 428.8  (338.9-529.4) | 0.118  (0.058-0.178) |
| Malawi | 40388.8  (31833.6-49236.4) | 487.6  (384.3-594.4) | 53272.8  (41875.7-65051.2) | 503.0  (395.4-614.2) | 0.325  (0.267-0.383) |
| Malaysia | 129978.4  (112054.0-153050.9) | 1208.5  (1041.9-1423.0) | 128023.5  (103292.8-154641.7) | 1248.4  (1007.2-1507.9) | 0.279  (0.104-0.454) |
| Maldives | 1430.6  (1151.6-1733.7) | 1047.6  (843.3-1269.6) | 1370.4  (1105.0-1677.8) | 1051.6  (848.0-1287.6) | 0.006  (-0.069-0.080) |
| Mali | 42719.9  (33253.3-53339.5) | 449.7  (350.1-561.5) | 64605.4  (49944.9-78241.0) | 453.9  (350.9-549.7) | 0.122  (0.087-0.158) |
| Malta | 1063.8  (863.6-1293.2) | 1157.2  (939.4-1406.8) | 924.9  (745.4-1129.4) | 1101.6  (887.8-1345.2) | -0.482  (-0.548--0.416) |
| Marshall Islands | 189.3  (150.9-230.3) | 737.4  (588.0-897.5) | 172.2  (137.7-211.3) | 746.2  (596.9-915.6) | 0.110  (0.024-0.196) |
| Mauritania | 8328.0  (6273.8-10529.1) | 453.9  (341.9-573.9) | 10772.6  (8148.8-13576.7) | 461.9  (349.4-582.1) | 0.154  (0.115-0.192) |
| Mauritius | 4710.0  (3763.3-5714.7) | 1250.2  (998.9-1516.8) | 3662.5  (2896.4-4494.3) | 1233.5  (975.5-1513.7) | -0.145  (-0.218--0.071) |
| Mexico | 447866.2  (367972.6-530934.8) | 982.8  (807.5-1165.1) | 473878.7  (382825.3-573380.5) | 1098.2  (887.2-1328.8) | 1.254  (0.807-1.702) |
| Micronesia (Federated States of) | 371.9  (297.2-455.2) | 747.5  (597.4-914.9) | 308.9  (246.1-370.4) | 745.3  (593.9-893.8) | -0.064  (-0.139-0.012) |
| Monaco | 72.8  (59.0-89.6) | 1124.6  (910.7-1382.7) | 75.5  (61.6-91.9) | 1115.3(909.5-1357.5) | -0.081  (-0.098--0.064) |
| Mongolia | 8816.8  (7106.9-10548.1) | 853.8  (688.3-1021.5) | 11076.5  (8894.1-13581.2) | 843.8  (677.5-1034.6) | -0.043  (-0.255-0.170) |
| Montenegro | 1078.1  (869.3-1309.1) | 644.4  (519.6-782.5) | 968.0  (783.6-1174.3) | 643.7  (521.1-780.8) | -0.025  (-0.096-0.047) |
| Morocco | 160451.6  (128926.7-194270.4) | 1197.3  (962.1-1449.6) | 155986.5  (125044.8-189425.9) | 1207.7  (968.2-1466.6) | 0.125  (0.030-0.220) |
| Mozambique | 55290.0  (43666.2-68463.0) | 417.6  (329.8-517.1) | 74529.5  (58640.9-91833.8) | 419.8  (330.3-517.3) | 0.073  (0.046-0.101) |
| Myanmar | 297183.8  (240930.4-357110.9) | 1514.9  (1228.1-1820.3) | 312029.2  (252997.9-376495.9) | 1502.4  (1218.2-1812.9) | -0.110  (-0.140--0.081) |
| Namibia | 6794.8  (5349.3-8310.2) | 656.7  (517.0-803.2) | 7076.2  (5555.3-8746.9) | 660.5  (518.5-816.4) | 0.062  (0.009-0.114) |
| Nauru | 36.8  (29.8-44.9) | 726.4  (587.6-885.2) | 37.4  (29.8-45.7) | 729.0  (580.4-890.9) | 0.002  (-0.080-0.084) |
| Nepal | 100649.2  (81897.3-121358.0) | 776.3  (631.7-936.0) | 97115.8  (77684.6-117047.7) | 779.8  (623.7-939.8) | -0.064  (-0.184-0.056) |
| Netherlands | 40062.9  (32498.3-48704.5) | 1018.2  (825.9-1237.8) | 37556.8  (30447.3-45693.1) | 1017.5  (824.9-1237.9) | 0.008  (-0.022-0.038) |
| New Zealand | 16837.1  (13725.5-20674.6) | 1376.7  (1122.3-1690.5) | 17936.6  (14576.1-22052.2) | 1376.1  (1118.2-1691.8) | 0.030  (-0.026-0.086) |
| Nicaragua | 29036.2  (23616.9-35041.9) | 1117.1  (908.6-1348.1) | 28610.3  (23329.2-34475.8) | 1100.7  (897.5-1326.4) | -0.169  (-0.206--0.132) |
| Niger | 47352.1  (37419.9-57884.4) | 467.3  (369.3-571.2) | 74410.2  (58855.8-91400.6) | 477.4  (377.6-586.4) | 0.162  (0.116-0.207) |
| Nigeria | 587285.8  (480281.7-701023.9) | 634.4  (518.8-757.3) | 863234.3  (697758.0-1036917.9) | 675.6  (546.1-811.5) | 0.698  (0.466-0.931) |
| Niue | 4.0  (3.2-4.9) | 742.3  (592.2-906.8) | 3.9  (3.1-4.8) | 740.2  (586.8-915.7) | -0.040  (-0.121-0.040) |
| North Macedonia | 3455.7  (2799.0-4208.1) | 653.6  (529.4-796.0) | 2913.1  (2336.0-3548.0) | 652.3  (523.1-794.5) | 0.001  (-0.118-0.121) |
| Northern Mariana Islands | 143.5  (114.5-174.5) | 759.9  (606.4-923.6) | 116.0  (92.5-143.3) | 773.2  (616.7-955.6) | 0.093  (-0.074-0.261) |
| Norway | 14443.1  (11720.3-17426.2) | 1164.8  (945.2-1405.4) | 14598.0  (11836.0-17656.4) | 1175.9  (953.4-1422.2) | 0.062  (-0.071-0.194) |
| Oman | 27501.2  (22214.0-33741.3) | 2614.0  (2111.4-3207.1) | 37687.3  (30539.4-46548.9) | 2526.2  (2047.1-3120.2) | -0.318  (-0.563--0.072) |
| Pakistan | 911001.6  (727596.5-1105827.6) | 1020.5  (815.1-1238.8) | 1063369.5  (845973.8-1294783.6) | 973.6  (774.5-1185.4) | -0.471  (-0.634--0.308) |
| Palau | 39.8  (31.5-48.1) | 734.5  (581.0-886.7) | 33.1  (26.4-40.2) | 747.0  (596.1-907.1) | 0.159  (0.110-0.207) |
| Palestine | 29724.0  (23929.5-36278.2) | 1350.4  (1087.1-1648.1) | 32913.5  (26724.6-40184.8) | 1366.9  (1109.9-1668.9) | 0.113  (0.053-0.173) |
| Panama | 15159.2  (12364.1-18210.6) | 1132.8  (924.0-1360.9) | 17042.5  (13821.6-20298.0) | 1130.7  (917.0-1346.7) | -0.053  (-0.130-0.025) |
| Papua New Guinea | 38507.9  (31007.5-46705.8) | 1039.6  (837.1-1260.9) | 50313.2  (40481.0-60805.4) | 1027.0  (826.3-1241.2) | -0.146  (-0.196--0.096) |
| Paraguay | 48513.4  (39223.1-59121.8) | 1820.2  (1471.6-2218.2) | 48149.8  (38983.4-58724.8) | 1802.9  (1459.7-2198.9) | -0.098  (-0.136--0.059) |
| Peru | 176102.6  (142883.8-211032.9) | 1573.9  (1277.0-1886.1) | 191963.4  (156510.4-231458.9) | 1534.4  (1251.0-1850.1) | -0.271  (-0.310--0.232) |
| Philippines | 535706.6  (432025.5-647555.8) | 1246.0  (1004.9-1506.2) | 562946.2  (453044.8-686640.6) | 1257.2  (1011.8-1533.4) | 0.111  (0.078-0.143) |
| Poland | 56497.1  (46114.8-68647.7) | 682.1  (556.7-828.8) | 52908.6  (42909.4-64590.5) | 687.2  (557.4-839.0) | 0.072  (0.017-0.127) |
| Portugal | 25485.8  (20397.9-30926.3) | 1146.9  (918.0-1391.8) | 22070.1  (17741.9-26934.3) | 1151.4  (925.6-1405.2) | 0.028  (-0.033-0.089) |
| Puerto Rico | 10028.7  (7983.2-12325.4) | 967.9  (770.5-1189.6) | 6476.6  (5177.7-8011.9) | 991.1  (792.3-1226.0) | 0.183  (0.123-0.243) |
| Qatar | 4540.6  (3679.1-5552.4) | 1430.0  (1158.7-1748.6) | 8696.7  (6964.2-10659.7) | 1456.5  (1166.3-1785.3) | 0.205  (0.081-0.328) |
| Republic of Korea | 130007.6  (105682.0-158361.2) | 1129.4  (918.1-1375.8) | 94563.0  (76015.3-115308.1) | 1128.0  (906.8-1375.5) | 0.001  (-0.146-0.149) |
| Republic of Moldova | 9758.0  (7962.8-11782.4) | 1126.1  (918.9-1359.7) | 7857.4  (6330.6-9525.6) | 1128.5  (909.3-1368.1) | 0.050  (-0.037-0.136) |
| Romania | 28850.6  (23348.6-35222.1) | 641.5  (519.1-783.1) | 26188.0  (21018.7-32255.0) | 648.1  (520.2-798.2) | 0.081  (0.051-0.112) |
| Russian Federation | 344102.4  (281387.2-414174.7) | 1109.4  (907.2-1335.3) | 384381.1  (312783.0-467096.1) | 1137.6  (925.7-1382.4) | 0.247  (0.090-0.405) |
| Rwanda | 19926.3  (16077.2-24255.9) | 358.9  (289.5-436.8) | 23071.0  (18368.0-28178.9) | 358.4  (285.3-437.7) | 0.008  (-0.035-0.051) |
| Saint Kitts and Nevis | 145.2  (115.1-179.4) | 850.0  (673.9-1050.1) | 116.8  (92.2-142.0) | 830.8  (655.6-1009.8) | -0.266  (-0.321--0.211) |
| Saint Lucia | 471.6  (371.2-579.6) | 848.8  (668.2-1043.3) | 346.7  (273.1-426.2) | 826.2  (650.8-1015.9) | -0.319  (-0.393--0.245) |
| Saint Vincent and the Grenadines | 333.7  (264.3-409.9) | 841.7  (666.7-1033.7) | 282.0  (223.5-347.9) | 836.7  (663.0-1032.1) | -0.120  (-0.258-0.018) |
| Samoa | 684.1  (553.2-832.8) | 737.6  (596.5-897.9) | 742.7  (597.3-903.1) | 735.7  (591.7-894.7) | -0.054  (-0.116-0.008) |
| San Marino | 73.0  (59.4-88.9) | 1108.8  (901.6-1349.9) | 72.4  (58.3-87.6) | 1160.8  (934.4-1403.7) | 0.447  (0.426-0.469) |
| Sao Tome and Principe | 458.0  (361.9-562.5) | 489.8  (387.0-601.5) | 515.3  (407.3-628.4) | 506.2  (400.0-617.3) | 0.447  (0.426-0.469) |
| Saudi Arabia | 201819.5  (164972.4-242832.3) | 1968.8  (1609.3-2368.8) | 196664.6  (161298.2-235307.5) | 1940.8  (1591.8-2322.1) | -0.266  (-0.321--0.211) |
| Senegal | 30660.0  (24455.5-37440.3) | 453.0  (361.3-553.2) | 37313.5  (29536.3-45863.5) | 459.8  (364.0-565.1) | 0.123  (0.088-0.158) |
| Serbia | 14111.2  (11433.7-17273.2) | 649.5  (526.3-795.0) | 12333.4  (9866.8-15069.3) | 658.2  (526.5-804.2) | 0.139  (0.116-0.163) |
| Seychelles | 357.5  (287.0-433.8) | 1219.0  (978.8-1479.4) | 374.1  (299.5-455.8) | 1225.5  (981.2-1493.2) | 0.009  (-0.033-0.051) |
| Sierra Leone | 17148.3  (13444.0-20992.0) | 497.2  (389.8-608.7) | 22777.9  (17985.3-27932.1) | 500.5  (395.2-613.8) | 0.014  (-0.047-0.075) |
| Singapore | 15871.5  (12758.3-19161.6) | 1443.9  (1160.7-1743.3) | 14128.8  (11531.1-17321.7) | 1354.1  (1105.1-1660.1) | -0.590  (-0.871--0.309) |
| Slovakia | 7604.2  (6167.9-9214.3) | 636.7  (516.5-771.5) | 7179.3  (5768.8-8718.2) | 640.4  (514.6-777.6) | 0.049  (0.008-0.090) |
| Slovenia | 2482.3  (2014.0-3004.9) | 630.2  (511.3-762.9) | 2600.2  (2095.7-3189.6) | 642.3  (517.7-787.9) | 0.230  (0.146-0.313) |
| Solomon Islands | 2057.4  (1649.1-2527.9) | 734.2  (588.5-902.1) | 2455.0  (1980.5-2988.2) | 742.3  (598.8-903.5) | 0.063  (-0.031-0.156) |
| Somalia | 34741.8  (26726.1-43442.0) | 394.5  (303.5-493.3) | 49979.5  (39187.2-61772.7) | 390.9  (306.5-483.2) | -0.085  (-0.133--0.037) |
| South Africa | 124889.7  (99925.9-152907.3) | 631.1  (504.9-772.7) | 126562.1  (100750.0-154597.5) | 635.4  (505.8-776.2) | 0.083  (0.052-0.114) |
| South Sudan | 42200.6  (33980.4-50277.7) | 790.8  (636.8-942.2) | 42777.8  (34425.4-51387.0) | 779.6  (627.4-936.5) | -0.024  (-0.141-0.093) |
| Spain | 183165.2  (149160.9-220906.7) | 1983.0  (1614.9-2391.6) | 182710.8  (147999.1-219617.5) | 2074.5  (1680.4-2493.6) | 0.422  (0.314-0.530) |
| Sri Lanka | 100012.8  (79302.2-121110.9) | 1436.1  (1138.7-1739.1) | 100763.2  (80741.0-122862.6) | 1460.4  (1170.2-1780.6) | 0.146  (0.126-0.167) |
| Sudan | 209184.9  (169900.9-253774.8) | 1143.8  (929.0-1387.6) | 250611.6  (202261.2-307809.9) | 1172.4  (946.2-1440.0) | 0.192  (0.158-0.227) |
| Suriname | 1697.3  (1348.7-2090.5) | 871.3  (692.3-1073.1) | 1618.8  (1270.8-1985.1) | 852.7  (669.3-1045.6) | -0.261  (-0.387--0.136) |
| Sweden | 12849.7  (10094.6-16038.1) | 588.8  (462.5-734.9) | 14379.2  (11338.2-17808.7) | 595.3  (469.4-737.3) | 0.094  (0.033-0.155) |
| Switzerland | 18496.5  (14933.1-22436.4) | 1121.6  (905.5-1360.5) | 19399.8  (15694.6-23770.0) | 1100.6  (890.4-1348.6) | -0.182  (-0.243--0.121) |
| Syrian Arab Republic | 155117.0  (126494.1-186663.7) | 1496.1  (1220.1-1800.4) | 86395.3  (69241.9-105356.1) | 1588.1  (1272.8-1936.7) | 0.670  (0.548-0.792) |
| Taiwan (Province of China) | 28020.3  (22386.9-34568.3) | 533.3  (426.1-658.0) | 21146.5  (16936.9-25831.2) | 522.7  (418.7-638.5) | -0.224  (-0.377--0.071) |
| Tajikistan | 31115.1  (24956.4-38013.4) | 845.5  (678.1-1032.9) | 36791.7  (29215.6-45574.3) | 825.8  (655.8-1023.0) | -0.186  (-0.297--0.074) |
| Thailand | 267881.2  (214176.1-326387.4) | 1467.2  (1173.1-1787.6) | 199896.6  (159340.8-245994.4) | 1464.6  (1167.5-1802.4) | -0.036  (-0.057--0.015) |
| Timor-Leste | 8356.4  (6788.6-10129.6) | 1444.5  (1173.5-1751.0) | 9951.1  (8047.6-12095.1) | 1451.8  (1174.1-1764.6) | 0.028  (-0.042-0.098) |
| Togo | 16593.5  (13178.5-20229.4) | 490.3  (389.4-597.7) | 20805.6  (16411.9-25490.5) | 498.3  (393.1-610.5) | 0.106  (0.025-0.186) |
| Tokelau | 3.8  (3.0-4.6) | 764.9  (615.1-932.8) | 3.9  (3.1-4.8) | 764.8  (607.6-939.6) | -0.015  (-0.054-0.024) |
| Tonga | 294.2  (237.8-359.6) | 574.5  (464.3-702.2) | 285.4  (229.9-346.4) | 575.5  (463.7-698.7) | -0.072  (-0.192-0.048) |
| Trinidad and Tobago | 3290.8  (2555.4-4068.3) | 859.0  (667.1-1062.0) | 3125.3  (2475.8-3839.4) | 858.6  (680.2-1054.8) | -0.042  (-0.221-0.136) |
| Tunisia | 38836.5  (31722.7-47200.4) | 1080.8  (882.8-1313.5) | 38430.3  (31102.6-47244.8) | 1071.8  (867.4-1317.6) | -0.090  (-0.240-0.060) |
| Turkey | 275574.3  (221333.7-334195.2) | 1086.3  (872.5-1317.4) | 268242.7  (219201.1-327372.0) | 1085.5  (887.0-1324.7) | -0.037  (-0.118-0.044) |
| Turkmenistan | 21279.2  (17167.3-25921.5) | 1090.8  (880.1-1328.8) | 20814.8  (16627.2-25328.1) | 1061.7  (848.1-1291.9) | -0.219  (-0.294--0.145) |
| Tuvalu | 34.6  (27.9-41.7) | 759.4  (611.8-915.1) | 37.1  (29.9-44.7) | 755.0  (609.2-909.3) | -0.095  (-0.189--0.001) |
| Uganda | 69104.9  (54935.3-84297.6) | 347.1  (275.9-423.4) | 91297.4  (73014.7-110886.2) | 366.0  (292.7-444.5) | 0.524  (0.294-0.755) |
| Ukraine | 86275.4  (69960.2-103610.9) | 918.2  (744.6-1102.7) | 80723.1  (64317.2-97940.1) | 955.8  (761.6-1159.7) | 0.464  (0.340-0.588) |
| United Arab Emirates | 19518.1  (15848.9-23792.2) | 1423.7  (1156.0-1735.4) | 24523.4  (19762.5-29979.0) | 1446.7  (1165.9-1768.6) | 0.197  (0.013-0.380) |
| United Kingdom | 203975.8  (165250.0-245579.3) | 1351.1  (1094.6-1626.7) | 213079.0  (172532.2-258318.5) | 1361.1  (1102.1-1650.1) | 0.078  (-0.006-0.163) |
| United Republic of Tanzania | 114892.2  (89951.6-140246.7) | 465.0  (364.1-567.6) | 143669.6  (114089.0-175592.9) | 465.6  (369.7-569.0) | 0.015  (-0.007-0.038) |
| United States of America | 861294.8  (697719.5-1042248.5) | 1027.0  (831.9-1242.7) | 780794.1  (629657.2-949167.6) | 960.2  (774.4-1167.3) | -0.724  (-0.839--0.608)^*^ |
| United States Virgin Islands | 287.1  (228.9-351.7) | 942.4  (751.2-1154.4) | 173.4  (138.3-212.6) | 951.7  (758.9-1166.7) | 0.044  (-0.032-0.120) |
| Uruguay | 13948.3  (11246.0-17068.4) | 1377.1  (1110.3-1685.2) | 12465.1  (10126.7-15095.4) | 1379.9  (1121.0-1671.1) | -0.024  (-0.172-0.124) |
| Uzbekistan | 97775.4  (78897.3-119089.1) | 104569.7  (84077.9-127431.5) | 871.2  (703.0-1061.1) | 829.3  (666.7-1010.5) | -0.462  (-0.550--0.374) |
| Vanuatu | 813.2  (655.2-984.6) | 654.5  (527.4-792.5) | 968.7  (772.6-1178.2) | 657.0  (524.0-799.1) | 0.046  (0.016-0.076) |
| Venezuela (Bolivarian Republic of) | 103658.5  (84633.5-124547.8) | 976.3  (797.1-1173.1) | 85324.7  (70053.3-102241.3) | 974.9  (800.4-1168.2) | -0.139  (-0.317-0.040) |
| Viet Nam | 398024.4  (321957.4-477581.6) | 1288.7  (1042.4-1546.3) | 397120.8  (323627.4-475847.9) | 1256.0  (1023.5-1504.9) | -0.250  (-0.386--0.113) |
| Yemen | 153737.5  (121528.7-192231.6) | 1087.9  (860.0-1360.3) | 198924.0  (157169.8-249703.5) | 1138.5  (899.5-1429.2) | 0.467  (0.402-0.531) |
| Zambia | 29066.4  (22605.7-36471.5) | 365.3  (284.1-458.4) | 38890.6  (29635.8-48725.9) | 372.0  (283.5-466.1) | 0.199  (0.131-0.268) |
| Zimbabwe | 48169.8  (38534.6-58781.2) | 693.5  (554.8-846.3) | 56284.7  (45011.9-69055.1) | 704.9  (563.8-864.9) | 0.141  (0.095-0.186) |
