# Supplementary material for: Global, Regional, and National Burdens of Refraction Disorders in Children and Adolescents From 2010 to 2021
Source: J Ophthalmol. 2026 May 20;2026:5159332. doi: 10.1155/joph/5159332 (PMC13189494; doi:10.1155/joph/5159332)
Supplement: Supplementary file 1 — Supporting Information Supporting Figure 1. YLD rates of burden of refraction disorders in 21 regions (A) and 204 countries (B) by SDI in 2021. SDI = sociodemographic index. Supporting Figure 2. EAPC of prevalence rates (A) and YLD rates (B) of refraction disorders burden in 204 countries by SDI from 2010 to 2021. Supporting Table 1. Prevalence rate of refraction disorders by sex in global and 21 regions in 2010 and 2021. Supporting Table 2. YLDs rate of refraction disorders by sex in global and 21 regions in 2010 and 2021. Supporting Table 3. Prevalence rate of refraction disorders by age in global and 21 regions in 2010 and 2021. Supporting Table 4. YLDs rate of refraction disorders by age in global and 21 regions in 2010 and 2021. Supporting Table 5. Prevalence rate of refractive disorders and average annual percentage changes from 2010 to 2021 at 204 nations. Supporting Table 6 Years lived with disability of refractive disorders and estimated annual percentage changes from 2010 to 2021 at 204 nations. [file JOPH-2026-5159332-s001.zip › Supplementary Table 6.docx]

Supplementary Table 6 Years Lived with Disability of Refractive Disorders and Estimated Annual Percentage Changes from 2010 to 2021 at 204 nations

|  | YLDs | | | | |
| --- | --- | --- | --- | --- | --- |
|  | Case(n), 2010 | YLDs, 2010  (per 100 000 population) | Case(n), 2021 | YLDs, 2021  (per 100 000 population) | EAPC  2010-2021 |
| Nation |  |  |  |  |  |
| Afghanistan | 7748.7  (4813.9-11914.6) | 58.1  (36.1-89.3) | 10156.7  (6406.3-15674.3) | 57.1  (36.0-88.2) | -0.145  (-0.206--0.084) |
| Albania | 214.1  (130.7-337.8) | 23.6  (14.4-37.2) | 142.8  (88.8-223.8) | 23.1  (14.4-36.3) | -0.171  (-0.230--0.113) |
| Algeria | 6820.4  (4233.9-10614.4) | 49.6  (30.8-77.2) | 7910.1  (4809.4-12351.7) | 48.1  (29.2-75.1) | -0.309  (-0.536--0.081) |
| American Samoa | 6.9  (4.2-10.7) | 26.2  (16.1-40.6) | 5.1  (3.1-7.9) | 26.6  (16.3-41.2) | 0.120  (0.083-0.157) |
| Andorra | 6.3  (3.7-9.8) | 39.1  (23.3-60.6) | 5.9  (3.5-8.9) | 40.8  (24.4-62.2) | 0.448  (0.384-0.511) |
| Angola | 2143.9  (1286.2-3433.8) | 17.2  (10.3-27.6) | 3348.0  (1985.8-5387.4) | 17.9  (10.6-28.8) | 0.285  (0.164-0.406)^*^ |
| Antigua and Barbuda | 8.0  (4.8-12.6) | 28.3  (17.3-45.1) | 6.5  (4.0-10.4) | 27.8  (17.2-44.6) | -0.236  (-0.308--0.164) |
| Argentina | 7336.9  (4417.1-11276.1) | 51.9  (31.2-79.8) | 7143.5  (4161.1-11182.8) | 52.1  (30.3-81.6 | -0.006  (-0.158-0.146) |
| Armenia | 286.7  (172.9-445.1) | 33.7  (20.3-52.4) | 253.9  (157.3-394.4) | 33.3  (20.6-51.7) | -0.076  (-0.228-0.076) |
| Australia | 2595.7  (1570.4-4125.6) | 45.9  (27.7-72.9) | 2756.2  (1654.8-4350.2) | 44.2  (26.5-69.7) | -0.214  (-0.408--0.020) |
| Austria | 679.5  (417.1-1035.3) | 39.2  (24.1-59.7) | 674.2  (419.3-1035.1) | 38.4  (23.9-59.0) | -0.211  (-0.260--0.161) |
| Azerbaijan | 1060.8  (666.3-1635.0) | 34.1(21.4-52.6) | 1036.9  (658.6-1585.3) | 34.0  (21.6-52.0 | 0.023  (-0.109-0.154) |
| Bahamas | 34.4  (20.7-54.7) | 27.9  (16.8-44.4) | 33.0  (19.3-51.2) | 28.6  (16.8-44.4) | 0.233  (0.176-0.290) |
| Bahrain | 176.3  (108.5-274.4) | 54.2  (33.3-84.4) | 223.9  (137.4-341.7) | 55.5  (34.0-84.7) | 0.263  (0.200-0.325) |
| Bangladesh | 19567.8  (12207.8-30781.4) | 29.7  (18.5-46.7) | 18033.5  (11019.0-28585.8) | 29.6  (18.1-46.9) | 0.006  (-0.037-0.048) |
| Barbados | 12.0  (7.3-19.2) | 15.9  (9.7-25.4) | 10.7  (6.5-16.7) | 16.1  (9.7-25.1) | 0.109  (0.052-0.167) |
| Belarus | 627.0  (387.2-965.8) | 30.6  (18.9-47.1) | 626.4  (378.5-980.0) | 30.9  (18.7-48.4) | 0.150  (-0.070-0.370) |
| Belgium | 967.7  (609.5-1494.3) | 38.9  (24.5-60.0) | 996.6  (603.9-1568.0) | 39.2  (23.7-61.6) | 0.101  (0.017-0.186) |
| Belize | 43.0  (25.4-68.4) | 28.2  (16.7-44.8) | 48.2  (28.6-76.8) | 28.5  (16.9-45.5) | 0.051  (0.007-0.096) |
| Benin | 1018.7  (641.7-1548.8) | 18.8  (11.8-28.6) | 1395.2  (853.7-2200.4) | 18.6  (11.4-29.3) | -0.154  (-0.205--0.103) |
| Bermuda | 4.8  (2.9-7.3) | 33.5  (20.5-51.0) | 3.9  (2.3-6.1) | 33.7  (20.3-52.8) | 0.016  (-0.078-0.111) |
| Bhutan | 61.9  (38.0-94.0) | 21.3  (13.1-32.3) | 53.0  (32.1-81.0) | 20.8  (12.6-31.8) | -0.238  (-0.286--0.190) |
| Bolivia (Plurinational State of) | 1987.0  (1262.0-3074.7) | 44.7(28.4-69.1) | 2008.8  (1268.4-3155.5) | 44.2  (27.9-69.4) | -0.189  (-0.288--0.089) |
| Bosnia and Herzegovina | 201.4  (122.6-316.9) | 23.1  (14.0-36.3) | 153.4  (94.7-240.0) | 23.1  (14.2-36.1) | -0.026  (-0.088-0.037) |
| Botswana | 211.8  (128.5-328.3) | 24.0  (14.6-37.3) | 219.2  (132.2-347.6) | 23.9  (14.4-37.9) | -0.071  (-0.126--0.017) |
| Brazil | 45030.9  (27992.3-69513.3) | 67.3  (41.8-103.9) | 37912.5  (23782.9-58348.9) | 59.3  (37.2-91.3) | -1.455  (-2.002--0.906) |
| Brunei Darussalam | 53.4  (33.2-82.5) | 38.7  (24.0-59.8) | 49.3  (30.3-74.8) | 38.2  (23.5-58.0) | -0.114  (-0.158--0.070) |
| Bulgaria | 311.5  (190.6-484.6) | 22.5  (13.7-35.0) | 295.7  (177.8-459.0) | 22.8  (13.7-35.5) | 0.166  (0.100-0.233) |
| Burkina Faso | 1680.4  (1033.9-2554.5) | 17.3  (10.7-26.3) | 2214.7  (1356.3-3407.1) | 17.3  (10.6-26.6) | 0.015  (-0.054-0.084) |
| Burundi | 563.4  (345.8-862.7) | 10.7  (6.6-16.4) | 784.1  (498.5-1188.9) | 10.8  (6.9-16.3) | 0.105  (0.054-0.155) |
| Cabo Verde | 37.0  (23.2-57.2) | 16.4  (10.3-25.4) | 30.8  (19.4-47.7) | 16.0  (10.1-24.7) | -0.305  (-0.368--0.243) |
| Cambodia | 3621.8  (2178.1-5601.5) | 55.6  (33.4-86.0) | 3579.2  (2198.4-5476.6) | 54.0  (33.2-82.6) | -0.276  (-0.335--0.217) |
| Cameroon | 1937.7  (1224.3-2993.5) | 16.0  (10.1-24.6) | 2811.3  (1724.5-4276.8) | 16.6  (10.2-25.3) | 0.463  (0.330-0.596) |
| Canada | 2425.0  (1469.7-3783.8) | 30.5  (18.5-47.6) | 2492.0  (1518.5-3867.7) | 30.3  (18.5-47.0) | -0.062  (-0.094--0.030) |
| Central African Republic | 452.3  (268.5-720.6) | 18.0  (10.7-28.7) | 544.8  (323.5-864.9) | 18.8  (11.2-29.9) | 0.340  (0.230-0.450) |
| Chad | 1267.2  (792.9-1916.9) | 17.5  (11.0-26.5) | 1901.8  (1193.8-2958.7) | 17.4  (10.9-27.0) | -0.050  (-0.082--0.018) |
| Chile | 2595.4  (1579.2-4095.2) | 51.3  (31.2-80.9) | 2477.2  (1505.2-3910.7) | 50.6  (30.8-79.9) | -0.101(-0.232-0.030) |
| China | 87977.3  (55213.0-134499.2) | 27.3  (17.2-41.8) | 95764.4  (60895.9-147467.2) | 28.6  (18.2-44.1) | 0.800  (0.369-1.233) |
| Colombia | 6666.5  (3963.1-10102.4) | 40.2  (23.9-60.9) | 5767.4  (3467.9-8963.3) | 39.6  (23.8-61.6) | -0.183  (-0.244--0.122) |
| Comoros | 50.5  (30.7-80.7) | 16.4  (9.9-26.2) | 50.2  (31.2-77.7) | 16.0  (9.9-24.7) | -0.196  (-0.246--0.146) |
| Congo | 393.9  (232.0-631.5) | 19.0  (11.2-30.5) | 510.8  (299.3-825.4) | 20.5  (12.0-33.2) | 0.647  (0.488-0.806) |
| Cook Islands | 1.6  (1.0-2.5) | 25.0  (15.2-39.1) | 1.3  (0.8-2.0) | 24.8  (15.0-38.7) | -0.042  (-0.109-0.024) |
| Costa Rica | 570.3  (353.4-872.8) | 36.9  (22.9-56.5) | 499.1  (305.0-781.8) | 36.5  (22.3-57.1) | -0.161  (-0.234--0.088) |
| Croatia | 211.5  (131.3-330.4) | 22.7  (14.1-35.5) | 184.6  (114.3-290.6) | 22.9  (14.2-36.1) | 0.080  (0.059-0.101) |
| Cuba | 1228.2  (742.8-1938.0) | 44.1  (26.7-69.6) | 1038.3  (636.2-1601.8) | 43.2  (26.5-66.7) | -0.217  (-0.307--0.128) |
| Cyprus | 107.1  (66.1-167.3) | 39.4  (24.3-61.6) | 108.6  (67.7-171.1) | 38.1  (23.8-60.0) | -0.334  (-0.413--0.255) |
| Czechia | 570.3  (353.4-872.8) | 36.9  (22.9-56.5) | 499.1  (305.0-781.8) | 36.5  (22.3-57.1) | 0.188  (0.158-0.219) |
| Côte d'Ivoire | 2028.2  (1254.4-3156.0) | 18.1  (11.2-28.2) | 2586.9  (1590.8-3997.9) | 18.0  (11.1-27.8) | -0.073  (-0.117--0.030) |
| Democratic People's Republic of Korea | 1499.8  (895.9-2327.0) | 18.9  (11.3-29.3) | 1230.4  (720.6-1963.9) | 18.6  (10.9-29.7) | -0.168  (-0.213--0.123) |
| Democratic Republic of the Congo | 4936.6  (2918.8-7575.3) | 13.2  (7.8-20.2) | 6813.0  (4085.4-10654.0) | 14.2  (8.5-22.2) | 0.550  (0.420-0.680) |
| Denmark | 567.8  (343.1-890.6) | 42.0  (25.4-65.9) | 538.4  (331.4-850.2) | 41.6  (25.6-65.7) | -0.096  (-0.172--0.020) |
| Djibouti | 67.5  (42.1-106.2) | 16.8  (10.5-26.4) | 86.4  (53.6-133.8) | 16.4  (10.1-25.3) | -0.218  (-0.293--0.142) |
| Dominica | 6.8  (4.1-10.6) | 28.5  (17.1-44.5) | 5.6  (3.4-9.2) | 29.0  (17.5-47.2) | 0.155  (0.031-0.279) |
| Dominican Republic | 1576.6  (970.1-2480.4) | 40.3  (24.8-63.4) | 1502.4  (905.2-2331.2) | 38.6  (23.3-59.9) | -0.496  (-0.588--0.404) |
| Ecuador | 2176.9  (1340.5-3411.0) | 34.5  (21.3-54.1) | 2279.7(1420.3-3569.5) | 34.5  (21.5-54.0) | -0.041  (-0.088-0.005) |
| Egypt | 20120.6  (12367.7-30746.8) | 54.1  (33.2-82.6) | 25063.7  (15809.0-38582.9) | 53.8  (34.0-82.9) | 0.012  (-0.180-0.205) |
| El Salvador | 1219.0  (730.9-1932.5) | 45.9  (27.5-72.8) | 1059.4  (640.5-1653.8) | 44.5  (26.9-69.4) | -0.382  (-0.457--0.307) |
| Equatorial Guinea | 111.0  (66.9-179.0) | 18.9  (11.4-30.6) | 147.7  (88.8-237.5) | 19.2  (11.5-30.8) | 0.098  (-0.029-0.224) |
| Eritrea | 568.1  (351.8-862.5) | 20.2  (12.5-30.7) | 630.6  (404.7-965.8) | 19.6  (12.6-30.0) | -0.309  (-0.349--0.269) |
| Estonia | 58.3  (36.0-88.5) | 20.7  (12.8-31.5) | 59.3  (37.2-91.0) | 21.2  (13.3-32.5) | 0.178  (0.164-0.192) |
| Eswatini | 138.8  (84.5-220.3) | 24.9  (15.2-39.5) | 132.5  (81.8-206.6) | 24.8  (15.3-38.6) | -0.090  (-0.150--0.029) |
| Ethiopia | 12940.6  (8242.7-19683.5) | 26.3  (16.8-40.0) | 15092.5  (9755.8-23158.0) | 26.4  (17.1-40.5) | 0.068  (-0.040-0.176) |
| Fiji | 77.7  (47.1-119.7) | 23.2  (14.1-35.8) | 91.7  (53.9-144.6) | 26.2  (15.4-41.2) | 1.372  (0.896-1.850) |
| Finland | 476.4  (292.4-730.4) | 39.1  (24.0-59.9) | 456.1  (277.4-712.7) | 39.7  (24.2-62.1) | 0.196  (0.095-0.298) |
| France | 4948.2  (3077.0-7764.2) | 31.4  (19.5-49.3) | 4977.8  (3072.4-7724.1) | 31.6  (19.5-49.0) | 0.045  (-0.092-0.182) |
| Gabon | 132.7  (78.1-215.1) | 17.9  (10.5-29.0) | 152.4  (89.8-242.5) | 18.4  (10.9-29.3) | 0.198  (0.111-0.286) |
| Gambia | 181.6  (113.5-285.8) | 18.5  (11.6-29.1) | 241.0  (150.8-374.3) | 19.0  (11.9-29.6) | 0.258  (0.225-0.291) |
| Georgia | 351.7  (212.2-542.8) | 34.7  (20.9-53.5) | 317.9  (197.2-489.8) | 34.2  (21.2-52.7) | -0.125  (-0.274-0.024) |
| Germany | 5890.6  (3599.1-9184.8) | 38.9  (23.7-60.6) | 6034.7  (3722.2-9461.0) | 37.8  (23.3-59.3) | -0.279  (-0.309--0.248) |
| Ghana | 2934.5  (1807.9-4535.7) | 23.0  (14.2-35.5) | 3667.1  (2226.5-5630.1) | 22.5  (13.6-34.5) | -0.217  (-0.244--0.190) |
| Greece | 871.8  (520.5-1368.0) | 39.5  (23.6-61.9) | 760.4  (451.6-1191.7) | 39.9  (23.7-62.5) | 0.129  (0.103-0.156) |
| Greenland | 5.3  (3.2-8.2) | 30.5  (18.6-46.9) | 4.6  (2.8-7.1) | 29.9  (18.4-46.1) | -0.273  (-0.367--0.179) |
| Grenada | 10.6  (6.5-16.7) | 28.4  (17.2-44.5) | 8.5  (5.1-13.5) | 28.2  (17.0-44.7) | -0.165  (-0.249--0.081) |
| Guam | 15.6  (9.6-24.1) | 26.0  (16.0-40.2) | 12.4  (7.7-19.4) | 25.5  (15.8-40.0) | -0.178  (-0.248--0.108) |
| Guatemala | 3215.8  (1951.5-5029.6) | 46.4  (28.1-72.5) | 3069.6  (1865.9-4746.5) | 46.4  (28.2-71.8) | -0.036  (-0.076-0.005) |
| Guinea | 1058.3  (668.2-1629.6) | 18.9  (11.9-29.1) | 1377.3  (858.3-2167.8) | 18.5  (11.5-29.0) | -0.231  (-0.347--0.116) |
| Guinea-Bissau | 163.2  (97.6-252.7) | 18.6  (11.1-28.8) | 209.3  (127.2-320.2) | 18.7  (11.4-28.6) | 0.046  (-0.043-0.134) |
| Guyana | 92.1  (56.8-145.5) | 28.6  (17.7-45.3) | 76.7  (46.1-122.7) | 27.3  (16.4-43.7) | -0.554  (-0.648--0.460) |
| Haiti | 1337.9  (812.1-2085.8) | 27.8  (16.9-43.3) | 1535.6  (922.2-2369.6) | 27.4  (16.4-42.3) | -0.214  (-0.325--0.103) |
| Honduras | 1504.7  (919.0-2323.8) | 38.2  (23.3-58.9) | 1637.1  (975.4-2511.3) | 37.6  (22.4-57.7) | -0.171  (-0.221--0.121) |
| Hungary | 472.1  (290.2-730.1) | 22.7  (14.0-35.1) | 427.8  (261.6-658.7) | 22.8  (14.0-35.1) | 0.002  (-0.061-0.064) |
| Iceland | 33.3  (20.5-51.3) | 37.0  (22.8-57.0) | 32.8  (20.2-51.0) | 36.8  (22.6-57.2) | 0.060  (-0.022-0.141) |
| India | 170047.4  (106902.9-266121.1) | 33.1  (20.8-51.7) | 162343.2  (102323.2-253714.0) | 32.4  (20.4-50.7) | -0.089  (-0.248-0.069) |
| Indonesia | 32563.4  (20320.4-49875.2) | 35.6  (22.2-54.6) | 31966.7  (19924.0-49238.4) | 35.4  (22.1-54.6) | -0.061  (-0.111--0.012) |
| Iran (Islamic Republic of) | 17377.9  (10983.0-26450.3) | 69.0  (43.6-105.0) | 17269.9  (10920.7-26243.4) | 66.8  (42.2-101.5) | -0.393  (-0.557--0.228) |
| Iraq | 7423.2  (4596.3-11405.4) | 48.3  (29.9-74.2) | 8645.4  (5330.8-13628.8) | 49.0  (30.2-77.3) | 0.130  (0.041-0.219) |
| Ireland | 479.7  (293.6-748.3) | 37.8  (23.2-59.0) | 516.9  (314.0-805.3) | 39.1  (23.8-60.9) | 0.335(0.285-0.386) |
| Israel | 1062.9  (638.2-1662.0) | 37.8  (22.7-59.1) | 1279.6  (793.1-1987.0) | 37.9  (23.5-58.9) | 0.044  (-0.009-0.098) |
| Italy | 6462.3  (4018.6-9952.3) | 56.5  (35.1-87.0) | 6089.8  (3746.0-9465.1) | 58.1  (35.7-90.3) | 0.263  (0.227-0.298) |
| Jamaica | 292.4  (168.7-460.8) | 28.7  (16.6-45.3) | 232.2  (139.2-363.4) | 28.5  (17.1-44.6) | -0.180  (-0.276--0.085) |
| Japan | 7856.7  (4923.0-12098.6) | 33.3  (20.9-51.2) | 7122.9  (4482.6-10952.1) | 33.5  (21.1-51.6) | 0.105  (0.030-0.180) |
| Jordan | 1389.9  (864.9-2183.7) | 39.8  (24.8-62.6) | 2070.4  (1288.5-3249.1) | 42.0  (26.2-66.0) | 0.542  (0.442-0.642) |
| Kazakhstan | 1809.6  (1069.6-2799.4) | 33.4  (19.8-51.7) | 2228.8  (1386.7-3477.1) | 33.1  (20.6-51.7) | -0.029  (-0.107-0.049) |
| Kenya | 4221.5  (2709.2-6396.7) | 19.1  (12.3-29.0) | 4648.3  (2943.6-7039.2) | 18.9  (12.0-28.6) | -0.124  (-0.156--0.092) |
| Kiribati | 12.2  (7.1-19.1) | 24.8  (14.5-38.8) | 13.2  (7.7-21.0) | 24.6  (14.3-39.3) | -0.049  (-0.083--0.016) |
| Kuwait | 398.4  (244.0-628.5) | 46.1  (28.2-72.8) | 525.4  (327.2-811.8) | 48.0  (29.9-74.1) | 0.375  (0.268-0.483) |
| Kyrgyzstan | 558.8  (338.8-881.6) | 23.7  (14.4-37.4) | 658.7  (406.4-1042.4) | 23.3  (14.4-36.9) | -0.110  (-0.259-0.039) |
| Lao People's Democratic Republic | 876.9  (537.2-1356.4) | 28.9  (17.7-44.7) | 889.4  (544.1-1411.5) | 29.7  (18.2-47.2) | 0.161  (-0.169-0.491) |
| Latvia | 131.4  (80.4-205.7) | 30.6  (18.7-47.9) | 118.5  (73.2-185.7) | 30.6  (18.9-48.0) | -0.002  (-0.119-0.116) |
| Lebanon | 867.7  (556.2-1316.2) | 59.5  (38.1-90.2) | 982.6  (615.1-1477.9) | 59.0  (36.9-88.7) | -0.148  (-0.355-0.060) |
| Lesotho | 220.3  (135.9-342.2) | 24.9  (15.3-38.6) | 209.2  (129.3-326.4) | 24.9  (15.4-38.9) | -0.003  (-0.062-0.056) |
| Liberia | 398.2  (249.8-611.8) | 18.2  (11.4-28.0) | 518.0  (316.8-776.7) | 18.6  (11.4-27.8) | 0.142  (0.117-0.167) |
| Libya | 1216.4  (736.2-1893.1) | 51.5  (31.1-80.1) | 1115.6  (701.1-1736.3) | 53.4  (33.6-83.2) | 0.390  (0.277-0.503) |
| Lithuania | 211.3  (130.8-329.2) | 30.3  (18.8-47.2) | 160.1  (98.2-244.3) | 29.9  (18.3-45.6) | -0.124  (-0.172--0.076) |
| Luxembourg | 46.5  (28.5-72.7) | 39.1  (23.9-61.1) | 52.3  (32.2-81.2) | 38.7  (23.9-60.2) | -0.069  (-0.107--0.032) |
| Madagascar | 1910.6  (1219.6-2884.0) | 15.9  (10.2-24.1) | 2404.6  (1505.9-3679.1) | 16.1  (10.1-24.6) | 0.053  (-0.017-0.124) |
| Malawi | 1508.2  (901.6-2305.8) | 18.2  (10.9-27.8) | 1997.5  (1215.6-3097.9) | 18.9  (11.5-29.3) | 0.319  (0.254-0.384) |
| Malaysia | 4482.7  (2798.7-6835.9) | 41.7  (26.0-63.6) | 4406.7  (2676.9-6861.8) | 43.0  (26.1-66.9) | 0.270  (0.080-0.461) |
| Maldives | 47.8  (28.9-75.1) | 35.0  (21.2-55.0) | 45.4  (27.0-72.3) | 34.9  (20.7-55.5) | -0.059  (-0.140-0.023) |
| Mali | 1626.8  (1031.2-2552.1) | 17.1  (10.9-26.9) | 2415.1  (1501.0-3694.2) | 17.0  (10.5-26.0) | -0.058  (-0.108--0.008) |
| Malta | 37.0  (22.4-57.7) | 40.2  (24.3-62.8) | 32.1  (19.8-50.6) | 38.2  (23.6-60.3) | -0.475  (-0.539--0.412) |
| Marshall Islands | 6.4  (3.8-10.2) | 24.8(15.0-39.6) | 5.8  (3.5-9.0) | 25.0  (15.0-39.1) | 0.108  (0.031-0.185) |
| Mauritania | 314.8  (193.0-506.7) | 17.2  (10.5-27.6) | 406.3  (246.4-650.4) | 17.4  (10.6-27.9) | 0.127  (0.065-0.188) |
| Mauritius | 157.9  (95.6-244.8) | 41.9  (25.4-65.0) | 122.2  (73.9-189.6) | 41.2  (24.9-63.9) | -0.181  (-0.262--0.099) |
| Mexico | 17077.9  (10670.2-26250.6) | 37.5  (23.4-57.6) | 17783.9  (10953.5-27164.1) | 41.2  (25.4-63.0) | 1.078  (0.672-1.485) |
| Micronesia (Federated States of) | 12.5  (7.7-19.4) | 25.0  (15.5-39.1) | 10.3  (6.1-16.6) | 24.9  (14.6-40.1) | -0.100  (-0.178--0.021) |
| Monaco | 2.5  (1.5-4.0) | 39.1  (23.9-61.7) | 2.6  (1.6-4.0) | 38.6  (23.7-59.5) | -0.109  (-0.123--0.095) |
| Mongolia | 311.0  (193.9-485.6) | 30.1  (18.8-47.0) | 385.7  (235.0-601.7) | 29.4  (17.9-45.8) | -0.143  (-0.363-0.078) |
| Montenegro | 38.1  (23.1-60.1) | 22.7  (13.8-35.9) | 34.1  (21.0-54.3) | 22.7  (14.0-36.1) | -0.036  (-0.112-0.040) |
| Morocco | 5733.6  (3548.0-8932.5) | 42.8  (26.5-66.7) | 5559.0  (3429.7-8610.2) | 43.0  (26.6-66.7) | 0.089  (-0.006-0.184) |
| Mozambique | 2152.3  (1323.3-3261.8) | 16.3  (10.0-24.6) | 2874.8  (1753.3-4403.0) | 16.2  (9.9-24.8) | -0.009  (-0.052-0.034) |
| Myanmar | 10681.4  (6653.2-16447.3) | 54.4  (33.9-83.8) | 11013.7  (6799.0-17109.0) | 53.0  (32.7-82.4) | -0.257  (-0.285--0.229) |
| Namibia | 246.8  (150.0-385.9) | 23.8  (14.5-37.3) | 255.7  (162.3-406.6) | 23.9  (15.1-37.9) | 0.022  (-0.032-0.076) |
| Nauru | 1.2  (0.8-2.0) | 24.4(15.0-38.7) | 1.2  (0.7-2.0) | 24.3  (14.4-38.8) | -0.038  (-0.124-0.048) |
| Nepal | 3644.5  (2264.7-5592.6) | 28.1  (17.5-43.1) | 3446.3  (2107.1-5270.5) | 27.7  (16.9-42.3) | -0.207  (-0.311--0.104) |
| Netherlands | 1400.0  (865.2-2151.9) | 35.6  (22.0-54.7) | 1306.6  (798.6-2002.5) | 35.4  (21.6-54.3) | -0.020  (-0.062-0.022) |
| New Zealand | 579.8  (359.2-924.2) | 47.4  (29.4-75.6) | 616.7  (375.2-970.3) | 47.3  (28.8-74.4) | 0.004  (-0.048-0.055) |
| Nicaragua | 1049.0  (643.2-1630.9 | 40.4  (24.7-62.7) | 1029.1  (642.3-1616.3) | 39.6  (24.7-62.2) | -0.224  (-0.276--0.171) |
| Niger | 1792.3  (1109.3-2712.5) | 17.7  (10.9-26.8) | 2821.1  (1733.7-4326.2) | 18.1  (11.1-27.8) | 0.178  (0.130-0.225) |
| Nigeria | 23087.8  (14752.6-35183.1) | 24.9  (15.9-38.0) | 33364.6  (21127.2-51042.7) | 26.1  (16.5-39.9) | 0.520  (0.305-0.735) |
| Niue | 0.1  (0.1-0.2) | 24.8  (14.7-39.0) | 0.1  (0.1-0.2) | 24.6  (15.0-38.6) | -0.064  (-0.141-0.012) |
| North Macedonia | 121.9  (72.8-192.9) | 23.1  (13.8-36.5) | 102.8  (62.3-160.1) | 23.0  (14.0-35.9) | -0.018  (-0.138-0.101) |
| Northern Mariana Islands | 4.9  (2.9-7.8) | 26.0  (15.5-41.5) | 3.9(2.4-6.2) | 26.3  (16.1-41.4) | 0.076  (-0.102-0.253) |
| Norway | 500.3  (308.4-769.4) | 40.4  (24.9-62.1) | 505.0  (312.0-791.0) | 40.7  (25.1-63.7) | 0.064  (-0.048-0.176) |
| Oman | 959.9(590.7-1488.6) | 91.2  (56.1-141.5) | 1303.9  (812.8-2004.8) | 87.4  (54.5-134.4) | -0.394  (-0.646--0.141) |
| Pakistan | 32434.9  (20178.5-50278.0) | 36.3  (22.6-56.3) | 38136.3  (23875.5-59489.4) | 34.9  (21.9-54.5) | -0.380  (-0.552--0.208) |
| Palau | 1.3  (0.8-2.1) | 24.6  (14.7-39.3) | 1.1  (0.7-1.7) | 24.9  (15.0-38.4) | 0.112  (0.074-0.151) |
| Palestine | 1028.4  (640.8-1613.3) | 46.7  (29.1-73.3) | 1137.2  (687.5-1787.7) | 47.2  (28.6-74.2) | 0.092  (0.042-0.143) |
| Panama | 550.4  (346.0-859.7) | 41.1  (25.9-64.2) | 613.2  (378.5-937.6) | 40.7  (25.1-62.2) | -0.145  (-0.217--0.072) |
| Papua New Guinea | 1245.5  (732.1-1988.1) | 33.6  (19.8-53.7) | 1614.1  (955.6-2578.3) | 32.9  (19.5-52.6) | -0.211  (-0.262--0.161) |
| Paraguay | 1670.3(1045.2-2590.0) | 62.7  (39.2-97.2) | 1662.8  (1006.8-2606.9) | 62.3  (37.7-97.6) | -0.078  (-0.111--0.044) |
| Peru | 6301.4(3873.5-9724.3) | 56.3  (34.6-86.9) | 6800.5  (4237.4-10667.7) | 54.4  (33.9-85.3) | -0.377  (-0.431--0.323) |
| Philippines | 18068.4(11240.6-28346.1) | 42.0  (26.1-65.9) | 18944.4  (11745.5-29837.3) | 42.3  (26.2-66.6) | 0.087  (0.060-0.115) |
| Poland | 1993.6(1232.8-3127.0) | 24.1  (14.9-37.8) | 1866.9  (1159.4-2922.3) | 24.3  (15.1-38.0) | 0.059  (-0.007-0.124) |
| Portugal | 885.7(551.3-1400.1) | 39.9  (24.8-63.0) | 765.9  (466.6-1185.7) | 40.0  (24.3-61.9) | 0.022  (-0.025-0.070) |
| Puerto Rico | 357.4(220.3-555.0) | 34.5  (21.3-53.6) | 230.7  (139.0-357.0) | 35.3  (21.3-54.6) | 0.173  (0.095-0.252) |
| Qatar | 167.4(102.9-260.8) | 52.7  (32.4-82.1) | 319.5  (197.1-489.1) | 53.5  (33.0-81.9) | 0.192  (0.067-0.317) |
| Republic of Korea | 4643.4  (2804.8-7131.7) | 40.3  (24.4-62.0) | 3350.9  (2054.3-5258.0) | 40.0  (24.5-62.7) | -0.035  (-0.203-0.133) |
| Republic of Moldova | 344.8  (212.4-533.5) | 39.8  (24.5-61.6) | 274.3  (162.6-426.3) | 39.4  (23.4-61.2) | -0.043  (-0.134-0.048) |
| Romania | 1016.4  (656.6-1594.5) | 22.6  (14.6-35.5) | 921.6  (560.4-1455.4) | 22.8  (13.9-36.0) | 0.075  (0.046-0.104) |
| Russian Federation | 12089.3  (7532.4-18817.2) | 39.0  (24.3-60.7) | 13431.2  (8358.8-21100.9) | 39.7  (24.7-62.4) | 0.209  (0.050-0.368) |
| Rwanda | 771.7  (484.4-1169.0) | 13.9  (8.7-21.1) | 885.8  (555.2-1364.6) | 13.8  (8.6-21.2) | -0.103  (-0.150--0.055) |
| Saint Kitts and Nevis | 5.0  (3.0-7.9) | 29.0  (17.5-46.5) | 4.0  (2.4-6.2) | 28.3  (16.9-44.1) | -0.304  (-0.367--0.242) |
| Saint Lucia | 16.1  (9.6-26.5) | 29.0  (17.2-47.7) | 11.8  (7.0-18.7) | 28.2  (16.6-44.5) | -0.348  (-0.438--0.258) |
| Saint Vincent and the Grenadines | 11.4  (6.8-17.9) | 28.7  (17.1-45.3) | 9.6  (5.8-15.2) | 28.6  (17.2-45.2) | -0.106  (-0.241-0.028) |
| Samoa | 22.8  (13.9-35.7) | 24.6  (15.0-38.5) | 24.7  (14.9-39.8) | 24.5  (14.8-39.5) | -0.090  (-0.169--0.010) |
| San Marino | 2.5  (1.6-4.0) | 38.6  (23.7-60.5) | 2.5  (1.5-4.0) | 40.3  (24.6-63.4) | 0.447  (0.404-0.490) |
| Sao Tome and Principe | 17.2  (10.9-26.8) | 18.4  (11.7-28.7) | 19.3  (11.8-29.3) | 18.9  (11.6-28.8) | 0.261  (0.204-0.317) |
| Saudi Arabia | 7626.3  (4895.0-11560.7) | 74.4  (47.8-112.8) | 7383.3  (4605.9-11151.0) | 72.9  (45.5-110.0) | -0.187  (-0.230--0.145) |
| Senegal | 1232.4  (759.2-1893.8) | 18.2  (11.2-28.0) | 1474.8  (919.4-2231.6) | 18.2  (11.3-27.5) | -0.004  (-0.039-0.031) |
| Serbia | 497.4  (304.0-775.7) | 22.9  (14.0-35.7) | 435.9  (269.2-677.4) | 23.3  (14.4-36.1) | 0.138  (0.119-0.157) |
| Seychelles | 11.9  (7.1-18.5) | 40.7  (24.3-63.2) | 12.4  (7.7-19.5) | 40.8  (25.2-63.7) | -0.019  (-0.057-0.020) |
| Sierra Leone | 647.6  (398.0-994.6) | 18.8  (11.5-28.8) | 859.5  (522.8-1331.1) | 18.9  (11.5-29.3) | -0.006  (-0.061-0.049) |
| Singapore | 551.7  (334.5-871.3) | 50.2  (30.4-79.3) | 490.4  (301.1-756.7) | 47.0  (28.9-72.5) | -0.596  (-0.879--0.313) |
| Slovakia | 267.9  (162.2-418.8) | 22.4  (13.6-35.1) | 253.1  (155.6-391.0) | 22.6  (13.9-34.9) | 0.049  (0.006-0.093) |
| Slovenia | 87.6  (53.9-135.6) | 22.2  (13.7-34.4) | 91.4  (56.6-141.6) | 22.6  (14.0-35.0) | 0.208  (0.122-0.295) |
| Solomon Islands | 69.4  (41.7-109.7) | 24.8  (14.9-39.1) | 82.3  (50.6-127.4) | 24.9  (15.3-38.5) | 0.011(-0.081-0.102) |
| Somalia | 1312.3  (827.8-2024.2) | 14.9  (9.4-23.0) | 1876.1  (1156.5-2879.2) | 14.7  (9.0-22.5) | -0.157  (-0.215--0.100) |
| South Africa | 4545.9  (2871.1-7091.1) | 23.0  (14.5-35.8) | 4622.2  (2897.7-7218.2) | 23.2  (14.5-36.2) | 0.104  (0.082-0.125) |
| South Sudan | 1589.9  (989.6-2459.3) | 29.8(18.5-46.1) | 1591.2  (988.6-2441.2) | 29.0  (18.0-44.5) | -0.152  (-0.272--0.032) |
| Spain | 6657.0  (4075.8-10303.0) | 72.1  (44.1-111.5) | 6604.5  (4039.0-10223.8) | 75.0  (45.9-116.1) | 0.395  (0.286-0.505) |
| Sri Lanka | 3263.0  (1953.2-5144.4) | 46.9  (28.0-73.9) | 3275.9  (1995.6-5167.8) | 47.5  (28.9-74.9) | 0.123  (0.076-0.169) |
| Sudan | 7895.0  (4964.4-12053.8) | 43.2  (27.1-65.9) | 9328.1  (5903.8-14461.0) | 43.6  (27.6-67.7) | 0.062  (0.022-0.102) |
| Suriname | 57.0  (35.0-91.1) | 29.3  (18.0-46.8) | 54.6  (33.0-86.2) | 28.7  (17.4-45.4) | -0.253  (-0.382--0.124) |
| Sweden | 474.1  (298.5-751.9) | 21.7  (13.7-34.5) | 532.6  (327.7-838.3) | 22.1  (13.6-34.7) | 0.137  (0.071-0.204) |
| Switzerland | 642.0  (406.3-1002.9) | 38.9  (24.6-60.8) | 672.4  (419.0-1032.7) | 38.1  (23.8-58.6) | -0.173  (-0.236--0.111) |
| Syrian Arab Republic | 5787.0  (3620.5-8827.4) | 55.8  (34.9-85.1) | 3236.1  (2000.6-4962.3) | 59.5  (36.8-91.2) | 0.721  (0.591-0.852) |
| Taiwan (Province of China) | 935.4  (569.2-1457.7) | 17.8  (10.8-27.7) | 700.9  (410.8-1098.0) | 17.3  (10.2-27.1) | -0.293  (-0.456--0.130) |
| Tajikistan | 1067.8  (650.3-1661.4) | 29.0  (17.7-45.1) | 1259.9  (786.5-1981.1) | 28.3  (17.7-44.5) | -0.217  (-0.316--0.117) |
| Thailand | 8747.3  (5152.7-13788.6) | 47.9  (28.2-75.5) | 6505.8  (3951.4-10225.1) | 47.7  (29.0-74.9) | -0.067  (-0.089--0.045) |
| Timor-Leste | 271.1  (162.5-439.6) | 46.9  (28.1-76.0) | 324.2  (191.2-510.9) | 47.3  (27.9-74.5) | 0.067  (-0.011-0.145) |
| Togo | 624.9  (395.1-965.2) | 18.5  (11.7-28.5) | 774.7  (468.5-1199.5) | 18.6  (11.2-28.7) | 0.008  (-0.049-0.066) |
| Tokelau | 0.1  (0.1-0.2) | 25.7  (15.2-40.5) | 0.1  (0.1-0.2) | 25.5  (15.5-40.8) | -0.078  (-0.123--0.032) |
| Tonga | 10.0  (6.1-15.5) | 19.5  (11.9-30.2) | 9.6  (5.9-15.0) | 19.4  (12.0-30.3) | -0.142  (-0.227--0.057) |
| Trinidad and Tobago | 111.8  (66.8-175.4) | 29.2  (17.4-45.8) | 105.7  (65.0-166.5) | 29.0  (17.9-45.8) | -0.086  (-0.294-0.121) |
| Tunisia | 1405.5  (859.5-2135.0) | 39.1  (23.9-59.4) | 1381.5  (836.5-2164.0) | 38.5  (23.3-60.4) | -0.152  (-0.323-0.018) |
| Turkey | 10135.4  (6238.3-15326.1) | 40.0  (24.6-60.4) | 9837.4  (6031.6-15011.0) | 39.8  (24.4-60.7) | -0.053  (-0.126-0.020) |
| Turkmenistan | 735.1  (443.9-1145.3) | 37.7  (22.8-58.7) | 716.2  (441.5-1151.2) | 36.5  (22.5-58.7) | -0.272  (-0.345--0.199) |
| Tuvalu | 1.2  (0.7-1.8) | 25.5  (15.5-39.9) | 1.2  (0.8-1.9) | 25.1  (15.4-39.6) | -0.156  (-0.253--0.060) |
| Uganda | 2683.5  (1664.5-4045.6) | 13.5  (8.4-20.3) | 3448.4  (2096.7-5237.5) | 13.8  (8.4-21.0) | 0.329  (0.071-0.588) |
| Ukraine | 3061.3(1855.5-4852.8) | 32.6  (19.7-51.6) | 2862.1  (1726.7-4497.6) | 33.9  (20.4-53.3) | 0.480  (0.348-0.612) |
| United Arab Emirates | 723.9  (441.2-1109.9) | 52.8  (32.2-81.0) | 908.5  (563.4-1414.2) | 53.6  (33.2-83.4) | 0.205  (-0.010-0.422) |
| United Kingdom | 7024.7  (4330.9-10973.8) | 46.5  (28.7-72.7) | 7332.6  (4498.1-11467.7) | 46.8  (28.7-73.3) | 0.085  (-0.003-0.173) |
| United Republic of Tanzania | 4532.3  (2854.4-6993.1) | 18.3  (11.6-28.3) | 5483.3  (3392.6-8387.9) | 17.8  (11.0-27.2) | -0.281  (-0.318--0.244) |
| United States of America | 30091.9  (18841.9-46570.9) | 35.9  (22.5-55.5) | 27491.9  (17250.9-42527.1) | 33.8  (21.2-52.3) | -0.632  (-0.727--0.536) |
| United States Virgin Islands | 10.3  (6.2-16.2) | 33.7  (20.2-53.1) | 6.2  (3.8-9.8) | 33.8  (20.7-53.5) | 0.009  (-0.072-0.090) |
| Uruguay | 467.5  (287.2-717.9) | 46.2  (28.4-70.9) | 417.0  (252.1-657.3) | 46.2  (27.9-72.8) | -0.021  (-0.185-0.144) |
| Uzbekistan | 3394.4  (2083.1-5291.0) | 30.2  (18.6-47.1) | 3600.6  (2153.4-5779.2) | 28.6  (17.1-45.8) | -0.546  (-0.641--0.450) |
| Vanuatu | 27.0  (16.8-42.9) | 21.7  (13.5-34.6) | 32.1  (19.5-50.9) | 21.8  (13.3-34.5) | 0.028  (-0.008-0.064) |
| Venezuela (Bolivarian Republic of) | 3792.7  (2337.0-5944.3) | 35.7  (22.0-56.0) | 3114.5  (1897.6-4913.3) | 35.6  (21.7-56.1) | -0.185  (-0.391-0.021) |
| Viet Nam | 14015.8  (8586.0-21751.3) | 45.4  (27.8-70.4) | 13591.6  (8139.9-21389.6) | 43.0  (25.7-67.6) | -0.546  (-0.691--0.400) |
| Yemen | 5611.6  (3399.4-8697.2) | 39.7  (24.1-61.5) | 7280.2  (4442.8-11224.6) | 41.7  (25.4-64.2) | 0.518  (0.418-0.618) |
| Zambia | 996.3  (597.6-1558.8) | 12.5  (7.5-19.6) | 1329.9  (802.3-2153.2) | 12.7  (7.7-20.6) | 0.171  (0.103-0.239) |
| Zimbabwe | 1733.1  (1084.0-2721.0) | 25.0  (15.6-39.2) | 2021.5  (1262.1-3272.6) | 25.3  (15.8-41.0) | 0.128  (0.084-0.172) |
