# Supplementary figures and images for: Global, Regional, and National Burdens of Refraction Disorders in Children and Adolescents From 2010 to 2021
Source: J Ophthalmol. 2026 May 20;2026:5159332. doi: 10.1155/joph/5159332 (PMC13189494; doi:10.1155/joph/5159332)

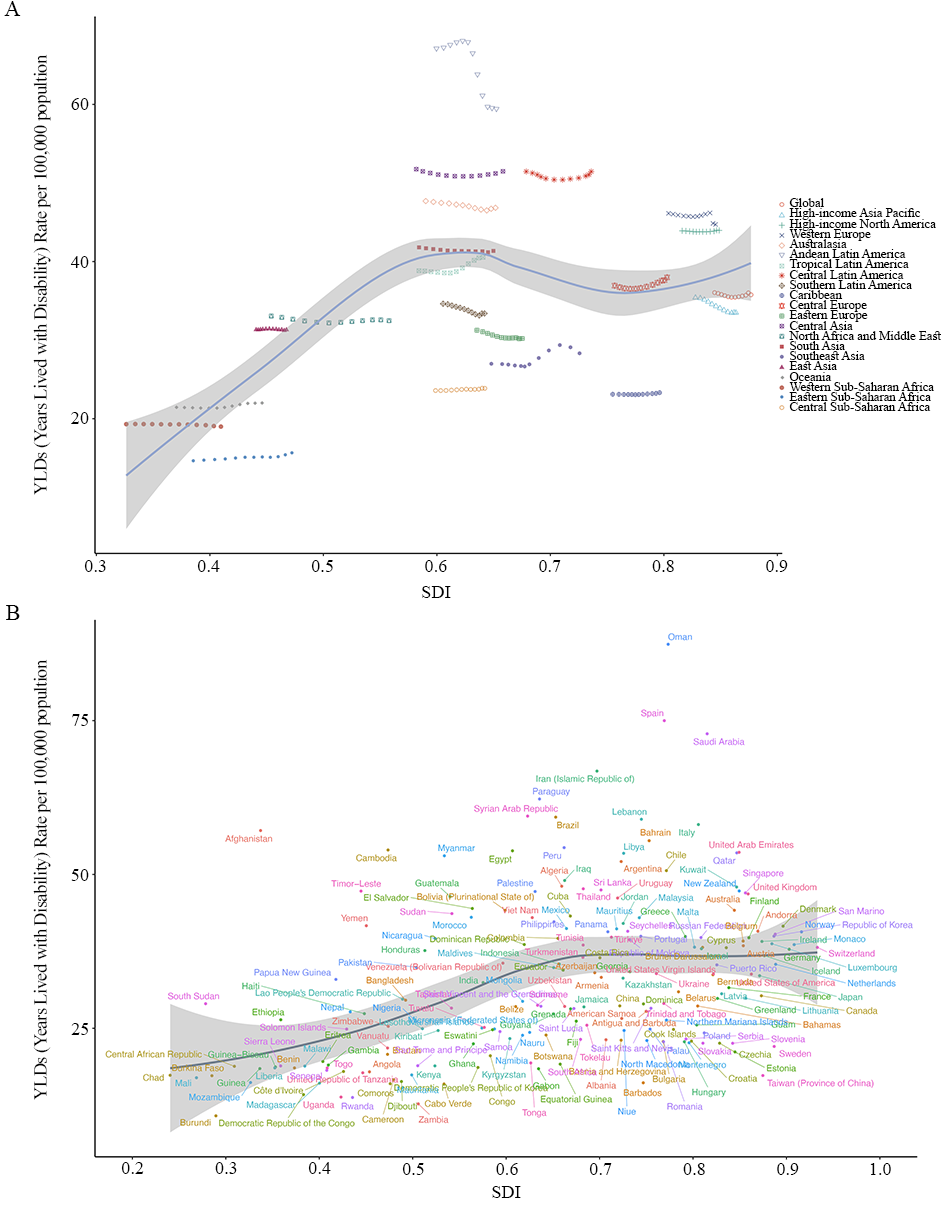

Supplement: Supplementary file 1 — Supporting Information Supporting Figure 1. YLD rates of burden of refraction disorders in 21 regions (A) and 204 countries (B) by SDI in 2021. SDI = sociodemographic index. Supporting Figure 2. EAPC of prevalence rates (A) and YLD rates (B) of refraction disorders burden in 204 countries by SDI from 2010 to 2021. Supporting Table 1. Prevalence rate of refraction disorders by sex in global and 21 regions in 2010 and 2021. Supporting Table 2. YLDs rate of refraction disorders by sex in global and 21 regions in 2010 and 2021. Supporting Table 3. Prevalence rate of refraction disorders by age in global and 21 regions in 2010 and 2021. Supporting Table 4. YLDs rate of refraction disorders by age in global and 21 regions in 2010 and 2021. Supporting Table 5. Prevalence rate of refractive disorders and average annual percentage changes from 2010 to 2021 at 204 nations. Supporting Table 6 Years lived with disability of refractive disorders and estimated annual percentage changes from 2010 to 2021 at 204 nations. [file JOPH-2026-5159332-s001.zip › Supplementary Figure1.tif]

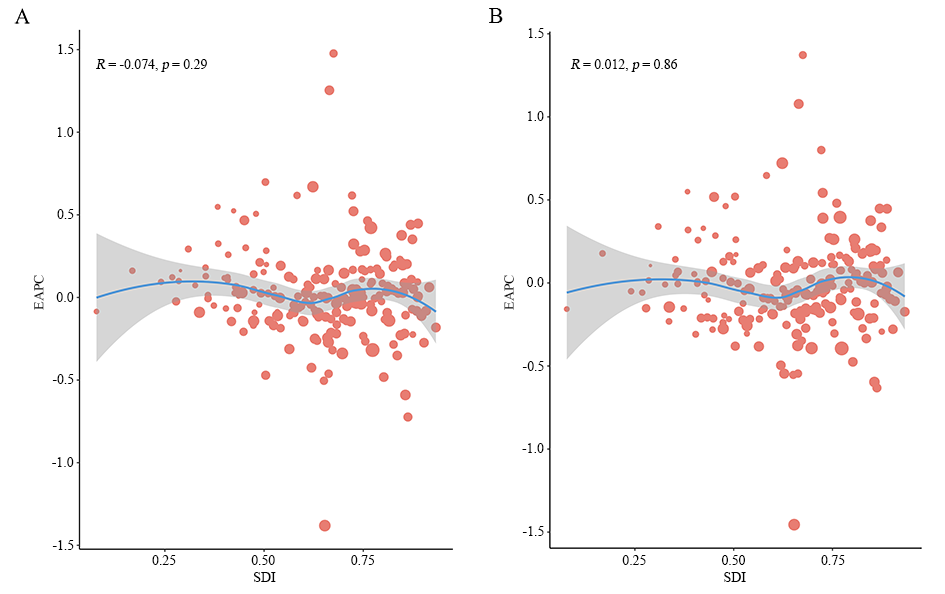

Supplement: Supplementary file 1 — Supporting Information Supporting Figure 1. YLD rates of burden of refraction disorders in 21 regions (A) and 204 countries (B) by SDI in 2021. SDI = sociodemographic index. Supporting Figure 2. EAPC of prevalence rates (A) and YLD rates (B) of refraction disorders burden in 204 countries by SDI from 2010 to 2021. Supporting Table 1. Prevalence rate of refraction disorders by sex in global and 21 regions in 2010 and 2021. Supporting Table 2. YLDs rate of refraction disorders by sex in global and 21 regions in 2010 and 2021. Supporting Table 3. Prevalence rate of refraction disorders by age in global and 21 regions in 2010 and 2021. Supporting Table 4. YLDs rate of refraction disorders by age in global and 21 regions in 2010 and 2021. Supporting Table 5. Prevalence rate of refractive disorders and average annual percentage changes from 2010 to 2021 at 204 nations. Supporting Table 6 Years lived with disability of refractive disorders and estimated annual percentage changes from 2010 to 2021 at 204 nations. [file JOPH-2026-5159332-s001.zip › Supplementray Figure2.tif]
